# Comparative Virulence and Genomic Analysis of *Streptococcus suis* Isolates

**DOI:** 10.3389/fmicb.2020.620843

**Published:** 2021-01-26

**Authors:** Tracy L. Nicholson, Ursula Waack, Tavis K. Anderson, Darrell O. Bayles, Sam R. Zaia, Isaiah Goertz, Mark Eppinger, Samantha J. Hau, Susan L. Brockmeier, Sarah M. Shore

**Affiliations:** ^1^National Animal Disease Center, Agricultural Research Service, United States Department of Agriculture, Ames, IA, United States; ^2^Oak Ridge Institute for Science and Education, United States Department of Energy, Oak Ridge, TN, United States; ^3^South Texas Center for Emerging Infectious Diseases, The University of Texas at San Antonio, San Antonio, TX, United States; ^4^Department of Biology, University of Texas at San Antonio, San Antonio, TX, United States

**Keywords:** *Streptococcus suis*, whole-genome sequencing, comparative genomics, virulence, mobile genetic elements, antimicrobial resistance, swine

## Abstract

*Streptococcus suis* is a zoonotic bacterial swine pathogen causing substantial economic and health burdens to the pork industry. Mechanisms used by *S. suis* to colonize and cause disease remain unknown and vaccines and/or intervention strategies currently do not exist. Studies addressing virulence mechanisms used by *S. suis* have been complicated because different isolates can cause a spectrum of disease outcomes ranging from lethal systemic disease to asymptomatic carriage. The objectives of this study were to evaluate the virulence capacity of nine United States *S. suis* isolates following intranasal challenge in swine and then perform comparative genomic analyses to identify genomic attributes associated with swine-virulent phenotypes. No correlation was found between the capacity to cause disease in swine and the functional characteristics of genome size, serotype, sequence type (ST), or *in vitro* virulence-associated phenotypes. A search for orthologs found in highly virulent isolates and not found in non-virulent isolates revealed numerous predicted protein coding sequences specific to each category. While none of these predicted protein coding sequences have been previously characterized as potential virulence factors, this analysis does provide a reliable one-to-one assignment of specific genes of interest that could prove useful in future allelic replacement and/or functional genomic studies. Collectively, this report provides a framework for future allelic replacement and/or functional genomic studies investigating genetic characteristics underlying the spectrum of disease outcomes caused by *S. suis* isolates.

## Introduction

*Streptococcus suis* is the leading bacterial pathogen affecting swine and contributes to significant economic losses to the swine industry worldwide (Feng et al., 2014; Segura et al., 2014a,b, 2017). *S. suis* causes a spectrum of clinical disease outcomes in pigs including pneumonia, endocarditis, septicemia, and meningitis. In addition, and perhaps more notably, *S. suis* is a zoonotic pathogen capable of causing diseases in humans, mainly arthritis, sepsis, meningitis, as well as streptococcal toxic shock-like syndrome (STSS) (Wertheim et al., 2009a,b; Feng et al., 2014; Segura et al., 2014a,b). Human infections have been either sporadic, and thought to be acquired from penetrating injuries associated with occupational exposure, or epidemic, and associated with consumption of raw or undercooked pork products, primarily in southeast Asia (Dutkiewicz et al., 2018).

Colonization and virulence mechanisms used by *S. suis* to cause disease in swine are not comprehensively characterized. Studies addressing specific virulence mechanisms used by *S. suis* have been confounded because different isolates cause a spectrum of disease outcomes ranging from lethal systemic disease to asymptomatic carriage (Segura et al., 2017). Factors generally regarded to contribute the most to virulence are the capsular polysaccharide (CPS), muramidase-released protein (mrp), extracellular protein factor (epf), and suilysin (sly) (Fittipaldi et al., 2012; Segura et al., 2014a,b, 2017). Individually, these factors have not been associated with the ability to cause invasive systemic disease and therefore virulence is thought to be multifactorial (Fittipaldi et al., 2012; Segura et al., 2017). Moreover, invasive clinical isolates obtained from people and pigs often do not harbor all of these factors (Fittipaldi et al., 2012; Feng et al., 2014; Segura et al., 2014a,b, 2017).

Mobile genetic elements (MGEs) are ubiquitous in bacteria and are the single most significant driver of gene transfer resulting in intra- and interspecies dissemination of antimicrobial resistance (AMR) and virulence determinants. The 89-kb pathogenicity island (89K PAI) carried by Chinese epidemic strains is an example of a *S. suis* MGE harboring factors contributing to a highly invasive phenotype (Li et al., 2011; Zhao et al., 2011). *S. suis* MGEs carrying AMR determinants for vancomycin, tetracycline, macrolide, aminoglycoside, and chloramphenicol resistance have been identified (Takamatsu et al., 2003; Palmieri et al., 2011a,b, 2012; Huang J. et al., 2016; Huang K. et al., 2016; Huang et al., 2018).

The majority of studies investigating *S. suis* isolate diversity rely on evaluating isolates by pulsed-field gel electrophoresis (PFGE), molecular serotyping, PCR typing of virulence markers, and/or determination of AMR profile (De Greeff et al., 2011; Zhang et al., 2011; Zheng et al., 2014; Prufer et al., 2019). While these methods consistently identify a small collection of virulence markers and AMR that *S. suis* isolates may harbor, they have yet to provide a complete evaluation of the genomic diversity, and what components of that diversity contributes to the virulence potential of *S. suis* isolates. A few recent studies have undertaken whole-genome sequencing (WGS) in combination with comparative genomic approaches to fully evaluate the genomic diversity and pathotype identification (Weinert et al., 2015; Estrada et al., 2019; Wileman et al., 2019). Although these studies relied on accurate clinical veterinary data, they did not directly test the virulence capacity of the *S. suis* isolates in pigs with similar health and immune status. Isolates from pigs with clinical signs and lesions consistent with *S. suis* disease are presumed to be virulent, while those obtained from the upper respiratory tract of healthy pigs are generally presumed to be avirulent. Unfortunately, this is an imprecise measure of virulence since conditions such as coinfection could enhance the apparent virulence of some isolates and existing immunity may mask the true virulence potential of others. The first objective of this study was to evaluate the virulence capacity of United States *S. suis* field isolates following intranasal challenge in swine. We choose to include eight isolates obtained from pigs exhibiting clinical disease consistent with *S. suis* disease and considered to be virulent, one *S. suis* strain isolated from the nasal cavity of an asymptomatic pig and considered to be non-virulent, and a reference isolate (P1/7) with known virulence. We hypothesized that the isolates considered to be virulent would exhibit different capacities to cause disease in swine if tested using a reference model free of confounding factors such as age, health status, differences in maternal antibody titers toward *S. suis*, and coinfection with other respiratory pathogens. After accurately establishing the virulence capacity of the isolates, our second objective was to obtain the closed-whole genome of each isolate and use comparative genomic analyses to evaluate the genetic and phenotypic attributes associated with swine-virulent phenotypes to link specific genes to *S. suis* disease outcomes.

## Materials and Methods

### *S. suis* Isolates and Culture Conditions

Nine *S. suis* isolates were evaluated in this study (SRD478, ISU2414, ISU2514, ISU2614, ISU2714, ISU2812, ISU2912, ISU1606, ISU2660) alongside a reference isolate (P1/7) with known virulence (Table 1). *S. suis* isolates were routinely grown at 37°C in Todd-Hewitt broth (Thermo Fisher Scientific Inc., Waltham, MA, United States) supplemented with 0.2% yeast extract (MilliporeSigma, St. Louis, MO, United States) (THY) and 5% filtered heat-inactivated horse serum (MilliporeSigma, St. Louis, MO, United States) (THY+) or on tryptic soy agar (TSA) containing 5% sheep blood (Becton, Dickinson and Co., Franklin Lakes, NJ, United States).

**TABLE 1:** Isolation site, clinical diagnosis, and reference for the nine United States *Streptococcus suis* isolates and reference isolate used in this study.

**Strain**	**Origin**	**Isolation Site**	**Diagnosis**	**References**
**P 1/7**	England	Blood	Septicemia/meningitis	Holden et al., 2009
**ISU2614**	US-IA	Brain	Pneumonia/Polyserositis	Hau et al., 2015
**ISU1606**	US-IA	Unknown	Unknown	Hau et al., 2015
**ISU2714**	US-IA	Brain	Pneumonia/meningitis	Hau et al., 2015
**ISU2660**	US-IA	Pleura	Septicemia/meningitis	Hau et al., 2015
**ISU2414**	US-IA	Pericardium	Pneumonia/Polyserositis	Hau et al., 2015
**ISU2514**	US-MT	Brain	Pneumonia/meningitis	Hau et al., 2015
**ISU2812**	US-IA	Brain	Pneumonia/meningitis	Hau et al., 2015
**ISU2912**	US-MI	Brain	Septicemia/meningitis	Hau et al., 2015
**SRD478**	US-IA	Nasal Cavity	Healthy	Hau et al., 2015

### Swine Virulence Experiments

Four-week-old Cesarean-derived, colostrum-deprived (CDCD) pigs (Struve Labs International, Manning, IA, United States) were housed in agricultural biosafety level 2 (ABSL2) containment and cared for in compliance with the Institutional Animal Care and Use Committee (IACUC) of the National Animal Disease Center (NADC). Each experimental group, consisting of 4-5 pigs, was housed in a separate isolation room in ABSL2 biocontainment facilities and was challenged with a separate *S. suis* isolate (Table 1). Experimental group size consisting of 4-5 pigs was based on a power analysis using analysis of variance (ANOVA) analysis with a minimal significance and statistical power of 0.05 and 0.80 respectively. For inoculum, *S. suis* isolates were grown on TSA containing 5% sheep blood (Becton, Dickinson and Co., Franklin Lakes, NJ, United States) at 37°C overnight, scraped from the plates and resuspended in phosphate buffered saline (PBS) to contain approximately 1 × 10^9^ colony forming units (CFU)/mL.

At approximately 8 weeks of age, nasal swabs were collected, placed in 1 mL phosphate buffered saline (PBS), and 100 μL of undiluted sample was immediately plated on TSA containing 5% sheep blood for determining the presence of *S. suis* and other common swine bacterial pathogens. Pigs were then intranasally challenged with 2 mL (1 mL per nostril) of approximately 1 × 10^9^ CFU/mL of each *S. suis* isolate in PBS. Following challenge, pigs were examined twice daily (morning and afternoon), except for an 8-h overnight period, for clinical signs of disease including lameness, lethargy, and neurological symptoms. When clinical signs were noted, a third check at 9 pm was performed. Pigs were euthanized if severe clinical presentation was observed (dyspneic, paddling and/or did not rise upon human entry into the pen). Ten days post-challenge, pigs failing to exhibit any signs of clinical disease were then comingled along with two CDCD naïve pigs, to evaluate transmission potential. As with the primary challenge, pigs were evaluated for clinical signs of disease for the next 15 days at which time all pigs were euthanized.

At necropsy gross lesions were recorded and samples were collected for culture: tonsil swabs, swab of serosa (pericardium, thoracic cavity, and abdominal cavity), joint fluid from the hock joint (or other affected joint), cerebrospinal fluid (CSF), lung lavage, and serum. Samples were collected in 2 mL PBS, except lung lavage, where 50 mL of PBS was instilled into the lung just above the tracheal bifurcation and aspirated with a pipette. 100 μL of all of the samples were plated on TSA containing 5% sheep blood for *S. suis* culture, which was confirmed by colony morphology and species-specific PCR. To determine the *S. suis* strain isolated from necropsy samples collected from pigs post-comingling, genomic DNA was extracted from isolated colonies and used as the template in PCR amplicon sequencing targeting the *aroA* and *mutS* MLST alleles. DNA sequence analysis was performed on a 557-bp DNA fragment within the *aroA* gene amplified by PCR using the primers 5′-TTCCATGTGCTTGAGTCGCTA-3′ and 5′-ACGTGACCTACCTCCGTTGAC-3′ and on a 716-bp DNA fragment within the *mutS* gene amplified by PCR using the primers 5′-GGCACAACAGTATATCGACACACTTG-3′and 5′-CCTGACGATTCTCAATCCGCTTAA-3′.

### Ethics Statement

Animal studies were conducted in accordance with the recommendations in the Guide for the Care and Use of Laboratory Animals of the National Institutes of Health. The animal experiments were approved by the USDA-National Animal Disease Center’s Institutional Animal Care and Use Committee (protocol #2724).

### Whole-Genome Sequencing, Assembly, and Annotation

Genomic DNA from *S. suis* isolates (Table 1) was extracted using a MasterPure Gram-positive bacterial DNA purification kit (Lucigen Corporation, Middleton, WI, United States) with the following modifications to the manufacturer’s instructions to prevent degradation. 1 mL from overnight grown liquid cultures was pelleted (5,000 × *g* for 5 min), the supernatant was removed, and the pelleted cells were resuspended in 300 μL lysis buffer consisting of 2% SDS (Thermo Fisher Scientific Inc., Waltham, MA, United States), 0.25 M EDTA (Thermo Fisher Scientific Inc., Waltham, MA, United States), and 30% Proteinase K (Roche, Mannheim, Germany). The suspension was incubated for 3 h at 55°C. 300 uL of Gram Positive Lysis solution from the MasterPure Gram-positive bacterial DNA purification kit was then added and the suspension was incubated for 30 min at 70°C. Samples were then placed on ice for 5 min, 350 uL of MPC Protein Precipitation Reagent from the MasterPure Gram-positive bacterial DNA purification kit was added and the DNA precipitation instructions provided by the manufacturer’s protocol were followed.

Whole genome sequencing was performed using the Pacific Biosciences (PacBio) platform. Library preparation for PacBio sequencing was performed following the PacBio 10-kb insert library preparation protocol available online at http://www.pacb.com/wp-content/uploads/2015/09/Procedure-Checklist-10-kb-Template-Preparation-and-Sequencing.pdf. The 10 kb library for each strain was sequenced using the PacBio RSII platform with two SMRT cells for each isolate. Closed whole-genome assemblies for isolates ISU1606, ISU2660, ISU2414, ISU2515, ISU2614, ISU2714, ISU2812, and SRD478 were generated using the hybrid assembler Unicycler v. 0.4.4 (Wick et al., 2017) software along with both the PacBio sequencing reads and the Illumina MiSeq platform paired-end sequencing reads, which were previously used to obtain draft assemblies for the 9 United States isolates (Hau et al., 2015). Closed whole-genome assembly for isolate ISU2912 was generated using PacBio smrtanalysis v. 2.3.0^1^, CANU v. 1.3 (Koren et al., 2017), and GARM v. 0.7.5 (Soto-Jimenez et al., 2014) along with PacBio sequencing reads and the Illumina MiSeq platform paired-end sequencing reads (Hau et al., 2015). Default parameters were used for all software. Final annotations were completed using NCBI’s Prokaryotic Genome Annotation Pipeline (PGAP) (Tatusova et al., 2016). Accession numbers and genome statistics are summarized in Table 4.

**TABLE 4:** *S. suis* genome sequences general summary.

	**P1/7^*a*^**	**ISU2614**	**ISU1606**	**ISU2714**	**ISU2660**	**ISU2514**	**ISU2414**	**ISU2812**	**ISU2912**	**SRD478**
**Serotype**	2	2	2	2	2	2	2	19	NCL1-3	undefined
**MLST sequence type (ST)**	1	28	1	1	787	25	620	76	786	785
**Chromosome Size (bp)**	2,007,491	2,163,384	2,073,988	2,063,877	2,182,487	2,248,415	2,222,543	2,563,853	2,720,381	2,063,454
**G + C Content (%)**	41.30%	41.20%	41.20%	41.20%	41.10%	41.10%	41.10%	41.10%	41.30%	41.20%
**Total CDSs**	1908	2164	2025	2006	2141	2243	2203	2434	2667	1990
**Pseudogenes^*b*^**	82	100	66	63	97	127	116	178	159	96
**Functional CDSs^*c*^**	1826	2064	1959	1943	2044	2116	2087	2256	2508	1894
**rRNA (16S-23S-5S)**	4-4-4	4-4-4	4-4-4	4-4-4	4-4-4	4-4-4	4-4-4	4-4-4	4-4-4	4-4-4
**tRNA**	56	56	56	56	56	57	56	57	58	56
**Plasmid**	0	1	0	0	0	1	0	1	0	4
**Accession Numbers^*d*^**	AM946016	CP031377 CP031378	CP030017	CP030022	CP031379	CP030020 CP030021	CP030023	CP030015 CP030016	CP030030	CP030010 CP030011 CP030012 CP030013 CP030014
**Virulence Categorization**	highly virulent	highly virulent	highly virulent	moderately virulent	moderately virulent	moderately virulent	non-virulent	non-virulent	non-virulent	non-virulent

*^*a*^Based on details provided by Holden et al. (2009). ^*b*^Pseudogenes are CDSs identified in the annotation as containing either a frameshift, internal stop codon, or an incomplete predicted protein sequence. ^*c*^Functional CDSs are based on the total CDSs identified minus pseudogenes. ^*d*^Accession numbers provided are for chromosome followed by plasmid(s). Font color used for isolate names is reflective of virulence categorization (green: highly virulent; blue: moderately virulent; black: non-virulent).*

### Comparative Genomic Analysis

Closed whole-genomes were compared with Blast Ring Image Generator (BRIG) (Alikhan et al., 2011) and progressiveMauve (Darling et al., 2010). Prophage regions were determined using PHASTER (Arndt et al., 2016).

Genomic islands were predicted using IslandViewer 4 (Bertelli et al., 2017). Insertion elements were determined using ISEscan in Galaxy (Afgan et al., 2018). Multilocus sequence type (MLST) was determined *in silico* based on the PCR typing scheme developed by King et al. (2002). Serotype was determined *in silico* based on PCR typing schemes described by Liu et al. (2013) for the classical serotypes and by Qiu et al. (2016) for the Novel CPS Loci (NCL) serotypes. Blastn analysis of the CPS genes was employed to determine sequence similarity to four non-typeable *S. suis* strains available in GenBank (accession numbers KX870048, KX870054, KX870055, and KX870059). Pan-genome analysis was performed using PanACEA^2^ (Clarke et al., 2018). For protein alignments, gene sequences were identified by blastn, translated, and subsequently aligned using default settings in MAFFT (Katoh et al., 2002). Percent identity for virulence-associated genes was determined for each isolate relative to the P1/7 ortholog with the following exceptions: strain P5/11/88 was used as *hylA* reference, strain 05ZHY33 was used as *revS* reference, strain GZ0565 was used as *stp* reference, and strain ZY05719 was used as reference for *vraR* and *vraS.* These exceptions were based on choosing a reference gene sequence in which functional characterization had previously been reported (*stp, vraR, and vraS*) (Zhu et al., 2011; Zhong et al., 2018) or due to annotation of the P1/7 gene as a pseudogene (*hylA* and *revS*). Heatmap generation and hierarchical clustering analysis was performed using MeV 4.8.1 (Saeed et al., 2006).

### Phenotypic and Genomic AMR Analysis

Phenotypic antibiotic resistance was determined using the broth microdilution method by Iowa State University Veterinary Diagnostic Laboratory following standard operating procedures. Minimum inhibitory concentrations (MICs) were determined for each isolate using the Trek BOPO6F plate (Thermo Fisher Scientific Inc., Oakwood Village, OH, United States) with *Streptococcus pneumoniae* ATCC 49619 and *Mannheimia haemolytica* ATCC 33369 (ATCC, Manassas, VA, United States) serving as the quality control strains. MICs were evaluated in accordance with Clinical Laboratory Standards Institute (CLSI) recommendations based on VET01-A4 and VET01S to give resistance interpretations (Supplementary Table 3; Clinical and Laboratory Standards Institute, 2013, 2015).

ResFinder 3.0 from the Center for Genomic Epidemiology^3^ and the Comprehensive Antibiotic Resistance Database (CARD)^4^ were employed for AMR genomic element identification. Genomes submitted to ResFinder 3.0 were evaluated for AMR determinants using standard default parameters of a threshold ID of 90% and a minimum length of 60%. To ensure that all AMR determinants had been identified, a second less stringent set of parameters of a threshold ID of 60% and a minimum length of 20% were used. The second and less stringent search did not result in identifying any additional AMR determinants. Genomes submitted to CARD were evaluated for AMR determinants using the criteria “default – perfect and strict hits only.”

### Cell Surface Hydrophobicity

The relative surface hydrophobicity of *S. suis* isolates was determined by evaluating the change in absorbance following xylenes treatment, as previously described with modifications (Rosenberg et al., 1980). Briefly, *S. suis* isolates were suspended in PUM buffer (150 mM phosphate, potassium, urea, and magnesium, pH 7.1) to an absorbance at OD_400_ between 1.5 and 2.0. In a 10 mm round bottom test tube, 1.2 mL of bacterial suspension and 0.1 mL xylenes (Mallinckrodt Pharmaceuticals, Staines-upon-Thames, United Kingdom) were combined. The mixture was incubated at 37°C for 10 min. After incubation, the mixture was vortexed for 2 min and allowed to rest for 15 min at room temperature. Absorbance of the aqueous phase was determined at OD_400_ and used to calculate the change in OD_400_. Results are represented as a% OD_400_ retained after the assay. *S. suis* P1/7 Δ*cps2E*, a capsule deletion mutant, was used as a control (Faulds-Pain and Wren, 2013). At least three independent experiments were performed.

### Whole-Blood Sensitivity Assay

Overnight cultures started from a single colony were diluted to an OD_600_ of 0.05 in 5 mL THY + and grown at 37°C to an OD_600_ of approximately 0.5 (range 0.4 – 0.6). Bacterial cells were pelleted by centrifugation, washed with PBS, and resuspended in PBS. 1 mL of bacterial cells was added with 9 mL whole pig blood (collected morning of assay from non-infected conventional pigs housed on-site, NADC, Ames, IA, United States) or 9 mL PBS in a sterile flask. Flasks were then incubated for 1 h at 37°C with gentle agitation. Serial dilutions in PBS were then plated on blood agar plates to determine CFU counts, which were then used to calculate the ratio of treated bacteria (whole pig blood) compared to control (PBS) and these data were reported as the percent viable bacteria. At least three independent experiments were performed.

### Serum Sensitivity Assay

Overnight cultures started from a single colony were diluted to an OD_600_ of 0.05 in 5 mL THY + and grown at 37°C to an OD_600_ of approximately 0.5 (range 0.4 – 0.6). Bacterial cells were pelleted by centrifugation, washed with PBS, and resuspended in 5 ml PBS. To each well of a 96-well flat bottom plate (Corning, Sigma-Aldrich, Darmstadt, Germany) the following was added: 10 μL bacterial cells and 90 μL guinea pig serum (Quidel Corp., San Diego, CA, United States), or 90 μL heat-inactivated guinea pig serum, or 90 μL PBS. Plates were then incubated for 1 h at 37°C with gentle agitation. Serial dilutions in PBS were then plated on blood agar plates to determine CFU counts, which were then used to calculate the ratio of bacteria in treatment wells (guinea pig serum) compared to control wells (PBS) and reported as the percent viable bacteria. A serum-sensitive *Glasserella parasuis* H465 isolate was used as a quality control strain. At least three independent experiments with three technical replicates in each experiment were performed.

### Growth Kinetics

Kinetic growth of cultures was measured using a Bioscreen C Automated Microbiology Growth Curve Analysis System (Growth Curves USA, Piscataway, NJ, United States). Overnight cultures started from a single colony were diluted to an OD_600_ of 0.02 in 300 μL THY +. Plates were then incubated at 37°C for 24 h and the OD_600_ of each well was recorded every 15 min after 5 s of shaking. At least three independent experiments with three technical replicates were performed.

### Microtiter Plate Assay for Static Biofilm Formation

A static biofilm assay was performed using the standard crystal violet method as previously reported (Nicholson et al., 2013). Briefly, overnight cultures started from a single colony were diluted to an OD_600_ of 0.1 in THY+. Cultures (100 μL) were added to each well of a flat-bottomed 96-well plate (Corning, Sigma-Aldrich, Darmstadt, Germany) and incubated statically for 24 hrs at 37°C. After incubation, the OD_600_ was measured in all wells to determine growth. The supernatant and any unadhered bacteria were aspirated from all cultures and then the wells were washed three times with 200 μL PBS. The wells were stained with 150 μL 0.1% crystal violet for 10 min. Crystal violet dye was then removed and wells were washed three times with 200 μL PBS. After the plate had dried, 150 μL of 100% ethanol was added to the wells and allowed to incubate for 15 min. To determine biofilm levels, 125 μL was then transferred to a new plate and the absorbance was measured at OD_538_. At least three independent experiments with three technical replicates in each experiment were performed.

### Oxidative Stress Assay

Overnight cultures started from a single colony were diluted to an OD_600_ of 0.05 in 5 mL THY + and grown at 37°C to an OD_600_ of approximately 0.5 (range 0.4 – 0.6). Cultures were then divided into two 2.5 mL cultures. Hydrogen peroxide was added to the treated cultures to 10 mM final concentration and an equal volume of water was added to the untreated cultures. Cultures were incubated at 37°C for 15 min in a shaking incubator (250 rpm). After incubation, catalase was added to 10 μg/mL final concentration. Serial dilutions in PBS + 10 μg/mL catalase were then plated on blood agar plates to determine CFU counts, which were then used to calculate the ratio of bacteria in treated cultures (10 mM H_2_O_2_) and untreated cultures (H_2_O), and the results were reported as the percent viable bacteria. At least three independent experiments with three technical replicates in each experiment were performed.

### Hemolysis Assay

Overnight cultures started from a single colony were diluted to an OD_600_ of 0.05 in 5 mL complex media containing pullulan (CM-P) (Ferrando et al., 2014). Bacteria were pelleted by centrifugation, washed with PBS, resuspended in 5 mL PBS, and pelleted again to collect the supernatant. DTT was added to a final concentration of 5 mM and two-fold dilutions were prepared in CM-P + 5 mM DTT. 100 μL of the supernatant dilution was added to 100 μL 2% RBCs and incubated for 2 h at 37°C. Unlysed RBCs were pelleted by centrifugation (1500 × *g*, 10 min) and 100 μL of the supernatant transferred to a flat-bottomed 96-well plate (Corning, Sigma-Aldrich, Darmstadt, Germany) plate and absorbance was read at 540 nm. Lysis by 1% Triton X-100 was used as a reference for 100% lysis. Hemolytic activity was calculated as the highest dilution that induced at least 50% hemolysis. One Hemolytic unit was defined as the reciprocal of the highest dilution that induced at least 50% lysis of RBCs. At least three independent experiments with three technical replicates in each experiment were performed.

### Adherence to BEAS2B or J774.16 Cells

BEAS-2B (ATCC CRL-9609; human bronchial epithelial cell line) and J774A.1 (ATCC TIB-67; murine monocyte/macrophage cell line) cells were cultured in Dulbecco’s Modified Eagle Medium (DMEM) broth supplemented with 10% fetal bovine serum (FBS), 1% non-essential amino acids and 1% sodium pyruvate to 85% confluency or 2 × 10^5^ cells/well at 37°C with 5% CO2. Overnight cultures started from a single colony were diluted to an OD_600_ of 0.05 in 5 mL THY + and grown at 37°C to an OD_600_ of approximately 0.5 (range 0.4 – 0.6). Cultures were then pelleted by centrifugation and resuspended in DMEM at approximately 2 × 10^7^ CFU/mL (MOI = 100) in DMEM. Adhesion assays were carried out in triplicate and performed by removing growth medium from BEAS-2B or J774A.1 cells and then adding either medium alone or 1 mL of bacterial inoculum. Plates were centrifuged at 800 × g for 10 min and incubated at 37°C with 5% CO_2_ for 2 h. Wells were washed four times with 1 mL of the growth medium to remove non-adherent bacteria. BEAS-2B or J774A.1 cells were trypsinized using 0.5 mL of 0.125% trypsin and incubated for 10 min at 37°C. The total volume of each well was brought up to 1 mL with DMEM and cells were homogenized by pipetting. Serial dilutions were then plated on blood agar plates to determine CFU counts, which were then used to calculate the proportion of adherent bacteria, expressed as a percentage of the original inoculum. At least three independent experiments with three technical replicates in each experiment were performed.

### Nuclease Activity

Both cell-associated and secreted nuclease assays were performed using a previously reported method (Kiedrowski et al., 2011). Briefly, overnight cultures started from a single colony were diluted to an OD_600_ of 0.05 in 5 mL THY + and grown at 37°C to an OD_600_ of approximately 0.5 (range 0.4 – 0.6). Cultures were normalized to ensure an equivalent OD_600_ and pelleted by centrifugation to remove the supernatant. Bacterial cells were resuspended in fresh THY + and an equivalent volume of bacterial sample was incubated with 2 μM FRET substrate for 10 min. Nuclease activity was then quantified by measuring the difference in fluorescence (ex. 552 nm/em. 580 nm). Secreted nuclease activity assays were performed similarly except, after the bacterial cultures were pelleted by centrifugation, the supernatant was collected and used for measuring nuclease activity by incubation with 2 μM FRET substrate for 10 min, followed by measuring the difference in fluorescence (ex. 552 nm/em. 580 nm). At least three independent experiments with three technical replicates in each experiment were performed.

### Statistical Analysis

For each assay, we applied a one-way ANOVA with Tukey’s multiple comparisons test to assess the differences between groups performed using GraphPad Prism software (GraphPad, La Jolla, CA, United States). A *P*-value less than 0.05 was considered significant.

## Results and Discussion

### Swine Virulence Assessment

**FIGURE 1:**
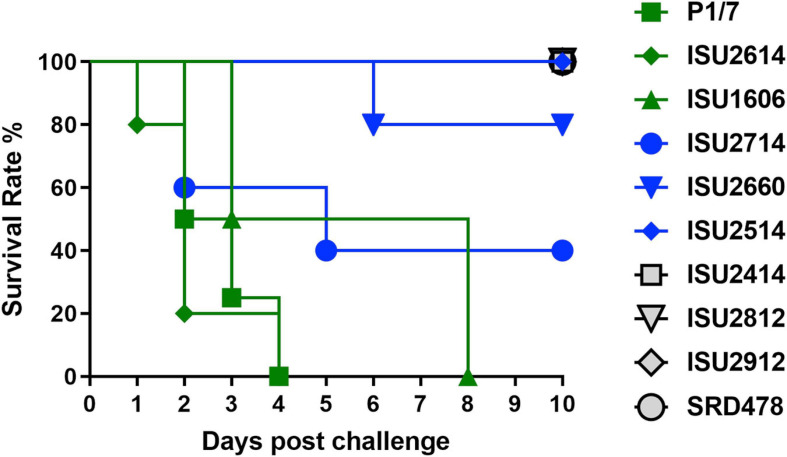
Survival rates of pigs post intranasal challenge. On day 0 groups of 4-5, 8-week-old CDCD pigs were intranasally inoculated with 2 mL (1 mL per nostril) of approximately 1 × 10^9^ CFU/mL of each *S. suis* isolate. The *x*-axis indicates days post-challenge, and the *y*-axis indicates percent survival.

The virulence capacity of nine United States *S. suis* field isolates, along with a highly virulent reference strain, P1/7, was assessed by evaluating morbidity and mortality following intranasal challenge of Cesarean-derived, colostrum-deprived (CDCD) pigs, a reference model free from many confounding factors such as health and immune status inherent in other model systems. *S. suis* isolates ISU2614 and ISU1606 exhibited high virulence, similar to strain P1/7, with all pigs in each of these groups developing systemic clinical disease within 8 days post-challenge (dpc) (Figure 1). Specifically, three of the pigs challenged with P1/7 exhibited clinical signs 1 day post-challenge. Two were euthanized 2 dpc, and the third was euthanized 4 dpc. One pig from this group began to exhibit clinical signs 2 dpc and was euthanized on the same day due to severe dyspnea. All pigs infected with P1/7 exhibited lethargy, while three pigs exhibited lameness on front and/or hind limbs, two pigs exhibited respiratory signs, and one pig exhibited neurological signs of disease (Table 2). At necropsy, *S. suis* was isolated from the CSF in all pigs infected with P1/7 (Table 3). *S. suis* was additionally isolated from a joint, or the serosa, or from serum for the majority, and from the lung for half of the pigs within this group (Table 3). One pig challenged with ISU2614 exhibited clinical signs 1 day post-challenge and was euthanized due to severe neurological signs (Figure 1). Three pigs infected with ISU2614 began to exhibit similar clinical signs 2 dpc and were euthanized on the same day due to either severe neurological signs or lameness due to swollen hocks (Figure 1). One pig challenged with ISU2614 exhibited clinical signs 3 dpc and was euthanized on day 4 post-challenge (Figure 1). The majority of pigs within this group exhibited lethargy and lameness on front and/or hind limbs, and neurological signs of disease, while 2 out of 5 pigs exhibited respiratory signs of disease (Table 2). At necropsy, *S. suis* was isolated from the serum and joint in all pigs infected with ISU2614, and from the serosa and CSF for the majority, and from the lung for 2 out of 5 pigs within this group (Table 3). Two of the pigs challenged with ISU1606 exhibited clinical signs 3 dpc and were euthanized, while the other pigs began to exhibit similar clinical signs 7 dpc and were euthanized on day 8 (Figure 1). All pigs within this group exhibited lethargy and lameness on front and/or hind limbs, two pigs exhibited neurological signs of disease, and one pig exhibited respiratory signs (Table 2). At necropsy, *S. suis* was isolated from the serum and serosa in all pigs infected with ISU1606, and from a joint for the majority, and from the lung and CSF for half of the pigs within this group (Table 3). Given that all pigs within these three groups rapidly developed clinical signs of systemic disease consistent with *S. suis* infection and were subsequently euthanized leaving no survivors, *S. suis* isolates ISU2614 and ISU1606 were categorized as highly virulent, along with strain P1/7.

**TABLE 2:** Frequency of clinical signs in *S. suis* infected pigs.

**Group**	**Respiratory**	**Neurologic**	**Lameness**
**P 1/7**	2/4^*a*^	1/4	3/4
**ISU2614**	2/5	4/5	4/5
**ISU1606**	1/4	2/4	4/4
**ISU2714**	0/5	3/5	3/5 + 1^*b*^
**ISU2660**	0/5	0/5	2/5
**ISU2514**	0/5	1/5 + 1^*c*^	2/5 + 1^*c*^
**ISU2414**	0/5	0/5	0/5
**ISU2812**	0/5	0/5	0/5
**ISU2912**	0/5	0/5	0/5
**SRD478**	0/5	0/5	0/5

*^*a*^Number of pigs that exhibited clinical signs out of number in challenge group. ^*b*^Transmitted to naïve pig and was isolated from joint. ^*c*^Transmitted to pig challenged with ISU2414 and was isolated from serum, joint, and CSF. Font color used for isolate names is reflective of virulence categorization (green: highly virulent; blue: moderately virulent; black: non-virulent).*

**TABLE 3:** Frequency of *S. suis* culture from various sites from pigs that died or were euthanized after demonstrating clinical signs.

**Group**	**Lung Lavage**	**Serum**	**Serosa**	**Joint**	**CSF**
**P 1/7**	2/4^*a*^	3/4	3/4	3/4	4/4
**ISU2614**	2/5	5/5	3/5	5/5	4/5
**ISU1606**	2/4	4/4	4/4	3/4	2/4
**ISU2714**	2/5	2/5	2/5	2/5 + 1^*b*^	3/5
**ISU2660**	0/5	0/5	0/5	2/5	0/5
**ISU2514**	0/5	0/5 + 1^*c*^	0/5	2/5 + 1^*c*^	1/5 + 1^*c*^
**ISU2414**	0/5	0/5	0/5	0/5	0/5
**ISU2812**	0/4	0/5	0/5	0/5	0/5
**ISU2912**	0/4	0/5	0/5	0/5	0/5
**SRD478**	0/4	0/5	0/5	0/5	0/5

*^*a*^Number of samples positive for *S. suis* out of number sampled. ^*b*^Transmitted to naïve pig and was isolated from joint. ^*c*^Transmitted to pig challenged with ISU2414 and was isolated from serum, joint, and CSF. Font color used for isolate names is reflective of virulence categorization (green: highly virulent; blue: moderately virulent; black: non-virulent).*

*Streptococcus suis* isolates ISU2714, ISU2660, and ISU2514 were moderately virulent following intranasal challenge. Three out of five pigs challenged with ISU2714 exhibited neurologic signs and lameness 2-4 dpc and were euthanized, while the other two pigs remained healthy and exhibited no signs of clinical disease (Figure 1 and Table 2). At necropsy, *S. suis* was isolated from the CSF in 3 out of 5 pigs infected with ISU2714, and from the joint, serosa, serum, and lung in 2 out of 5 pigs within this group (Table 3). Following challenge with ISU2660, one pig developed severe lameness 5 dpc and was euthanized on day 6 post-challenge (Figure 1 and Table 2). A second pig from this group also developed lameness 6 dpc, but subsequently recovered and remained healthy (Figure 1 and Table 2). The other 3 pigs challenge with ISU2660 remained healthy and exhibited no signs of clinical disease (Figure 1 and Table 2). At necropsy, *S. suis* was isolated from a joint in 2 out of 5 pigs infected with ISU2660 (Table 3). One pig challenged with ISU2514 developed lameness 8 dpc, but subsequently recovered and remained healthy (Figure 1 and Table 2). A second pig from this group exhibited delayed clinical signs of disease, which included lethargy and severe neurological signs and lameness, 13 dpc. The other 3 pigs challenged with ISU2514 remained healthy and exhibited no signs of clinical disease (Figure 1 and Table 2). At necropsy, *S. suis* was isolated from a joint in 2 out of 5 pigs infected with ISU2514 and from the CSF of a pig that exhibited delayed clinical disease (Table 3).

Following 10 dpc, pigs failing to exhibit any signs of clinical disease were subsequently comingled along with two CDCD naïve pigs, to evaluate transmission potential. During this period, a pig initially challenged with ISU2714 transmitted the bacterium to a naïve pig and ISU2714 was subsequently isolated from the joint of the contact pig. Additionally, a pig initially challenged with ISU2514 transmitted the bacterium to a pig initially challenged with ISU2414. ISU2514 was subsequently isolated from the joint, serum, and the CSF of the pig initially challenged with ISU2414 demonstrating transmission of isolate ISU2514. Collectively, the majority of the pigs initially challenged with isolates ISU2714, ISU2660, or ISU2514 remained healthy and exhibited no signs of clinical disease. Additionally, isolates ISU2714 and ISU2514 transmitted to cohorts leading to clinical signs of disease consistent with *S. suis* infection and were subsequently isolated from systemic sites. Because these isolates were capable of causing severe systemic disease but they were not uniformly lethal, isolates ISU2714, ISU2660, or ISU2514 were categorized as moderately virulent.

Following intranasal challenge with *S. suis* isolates ISU2414, ISU2812, ISU2912, or SRD478, all pigs in these groups remained healthy and exhibited no signs of clinical disease (Figure 1 and Tables 2, 3). Additionally, comingling with cohorts including naïve pigs failed to result in a transmission event leading to clinical signs of disease consistent with *S. suis* infection (Table 3). Based on these results, isolates ISU2414, ISU2812, ISU2912, and SRD478 were categorized as non-virulent.

While isolates ISU2414, ISU2812, and ISU2912 were categorized as non-virulent after internasal challenge of CDCD pigs, there were among the eight isolates obtained from pigs exhibiting clinical disease consistent with *S. suis* disease and were originally considered to be virulent (Table 1). In fact, ISU2414 was isolated from the pericardium and ISU2812 and ISU2912 were isolated from the brain (Table 1). It is possible that these isolates may have the capacity to cause systemic disease under different experimental conditions, such as circumventing the normal route of infection and using an intravenous challenge instead of an internasal challenge. Taken together, these results highlight the importance of utilizing a challenge method that closely resembles the normal route of infection along with a model free of confounding factors such as coinfections, which could enhance the apparent virulence of some isolates and existing immunity, which may mask the virulence potential of other isolates.

### Genome Assemblies, Features, and Comparisons

The complete genome assembly and annotation of all the *S. suis* isolates included in this study, along with a reference strain P1/7, is summarized in Table 4. The isolates harbored a variety of MLST sequence types (ST) with only ISU1606 and ISU2714 harboring the same ST as P1/7 (ST-1).

The chromosome size, total number of predicted protein coding sequences (CDSs), and G + C content of all the isolates was similar to P1/7, with the exception of isolates ISU2812 and ISU2912. ISU2812 contained a 2,563,863-bp chromosome, encoding 2,434 predicted CDSs, ISU2912 contained a 2,720,381-bp chromosome, encoding 2,667 predicted CDSs: the reference P1/7 contained a 2,007,491-bp chromosome, encoding 1,908 predicted CDSs (Table 4). These isolates were also observed to encode a greater number of predicted pseudogenes and functional CDSs compared to other isolates (Table 4). Previously, it has been hypothesized that virulence and zoonotic potential are correlated with a smaller genome size (Weinert et al., 2015). This correlative observation is consistent when comparing the genomic features of isolates ISU2812 and ISU2912 to P1/7, however, it is not consistent when comparing the genomic features of SRD478 to P1/7. Specifically, SRD478 contained a 2,063,454-bp chromosome, encoding 1,990 predicted CDSs, 96 predicted pseudogenes, and 1,894 functional CDSs, and is very similar to P1/7 genome features (Table 4).

**FIGURE 2:**
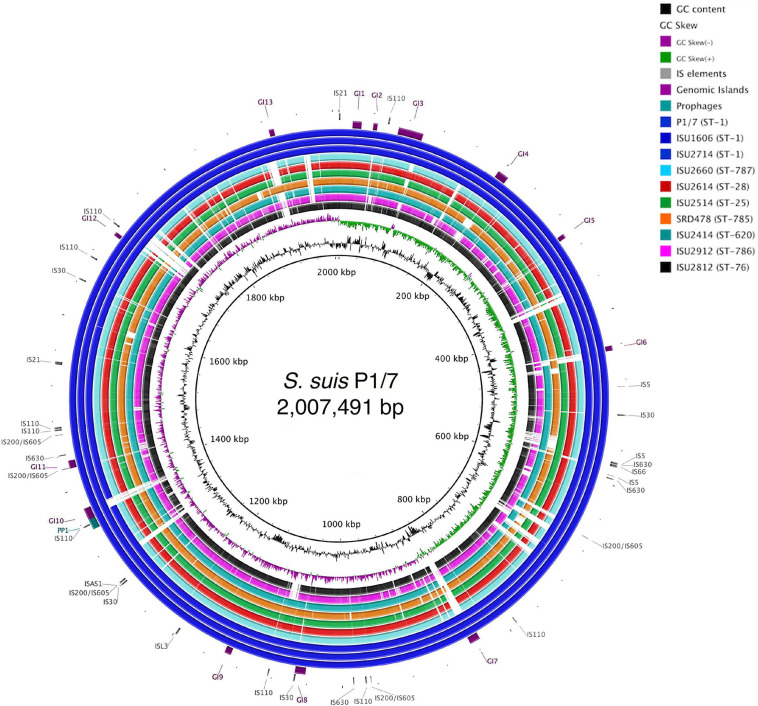
Genome visualization of *S. suis* chromosomes. Circular comparison of the of nine closed *S. suis* chromosomes referenced to the genome of ST-1 strain P1/7. Query genomes are and plotted from outermost to innermost ring and shown top to bottom in the legend. GC-content and -skew of the reference chromosome are depicted in the two innermost circles, respectively. Identified MGEs are shown as follows Genomic Islands (GI), ProPhage regions (PP), and Insertion elements (IS).

**FIGURE 3:**
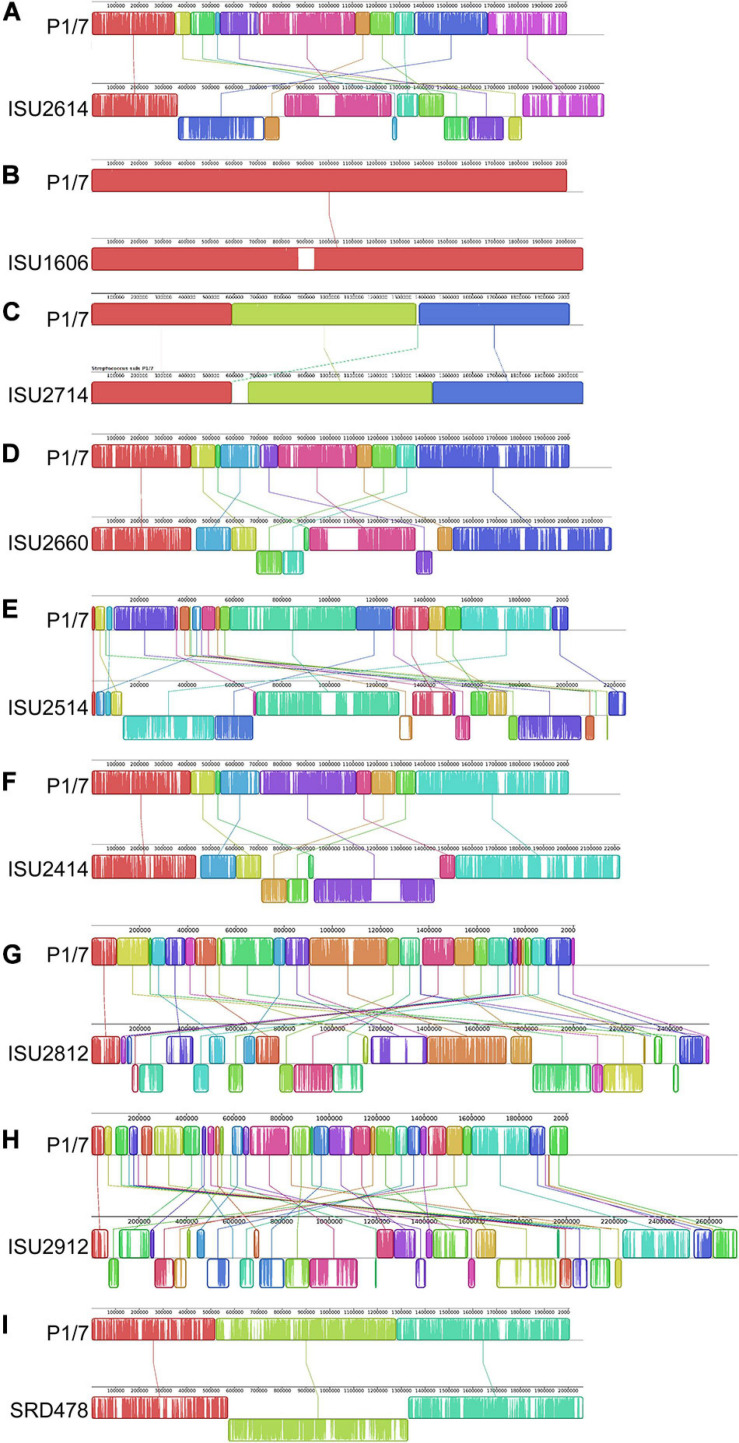
Comparison of the linear organization of *S. suis* chromosomes. Linear comparison of each closed *S. suis* genome to P1/7 (top), reference sequence. Locally collinear blocks (LCBs) representing regions of sequence alignment are shown as colored rectangles connected by lines. LCBs placed above the center line are in the same orientation as the reference; LCBs placed below the center line are in reverse orientation. Blank sections are regions of low sequence conservation. **(A)** P1/7 compared to ISU2614. **(B)** P1/7 compared to ISU1606. **(C)** P1/7 compared to ISU2714. **(D)** P1/7 compared to ISU2660. **(E)** P1/7 compared to ISU2514. **(F)** P1/7 compared to ISU2414. **(G)** P1/7 compared to ISU2812. **(H)** P1/7 compared to ISU2912. **(I)** P1/7 compared to SRD478.

The genomes for the 9 United States isolates were aligned and compared to the genome of P1/7 to examine genome architecture, chromosomal sequences and gene inventories, including mobile elements such as genomic islands, prophage regions, and insertion elements (Figure 2). A high degree of global synteny was observed among the ST-1 isolates P1/7, ISU1606, and ISU2714 (Figure 2). Genome synteny was further explored by comparing the linear organization of the chromosome of each *S. suis* isolate to P1/7. The highest degree of synteny was observed for isolate ISU1606 (Figure 3). A high degree of synteny within contiguously assembled regions of the genome was additionally observed for isolates SRD478 and ISU2714, with three and four, respectively, collinear regions of sequence alignment compared to P1/7 (Figure 3). An increased number of genome re-arrangements and inversions were observed for isolates ISU2414, ISU2660, and ISU2614 with nine, ten, and eleven, respectively, collinear regions of sequence alignment compared to P1/7 (Figure 3). Isolates ISU2514, ISU2812, and ISU2912 were found to contain a higher number of genome re-arrangements and inversions compared to P1/7 with 22, 29, and 38 respectively, collinear regions of sequence alignment (Supplementary Figure 1). Extensive genetic recombination among streptococcal species, including *S. suis* has been previously reported (Hanage et al., 2009; De Greeff et al., 2011; Zhang et al., 2011; Mostowy et al., 2014; Weinert et al., 2015). Therefore, the high number of genome re-arrangements and inversions compared to P1/7 observed for isolates ISU2514, ISU2812, and ISU2912 were not surprising. While ISU1606 and ISU2714 are both ST-1 isolates, the high degree of collinearity observed for SRD478 was unexpected given that it is a ST-785 isolate, which differs from ST-1 isolates at every allele.

To begin examining genomic differences that could have influenced the swine virulence capacity of the *S. suis* isolates, pan-genome analysis was used to investigate the genomic content among the *S. suis* genomes. A complete list of all the protein coding sequences (CDSs) identified within the *S. suis* genomes are summarized in Supplementary Table 1. Comparative analyses based on groups of orthologous proteins revealed 1,352 core CDSs that were shared among all ten isolates, 2,514 accessory CDSs that were present in 2-9 isolates, and 1,468 CDSs unique to single isolates (Supplementary Table 1). In addition to the high number of unique CDSs compared to the number of core CDSs (1,468 versus 1,352), wide variation in the number of unique CDSs harbored by single isolates was also observed. No unique CDSs were found in isolate ISU2714. Isolates P1/7 and ISU1606 both contained five unique CDSs (Supplementary Table 1). In contrast, isolate ISU2912 was found to contain 693 unique CDSs and isolate ISU2812 was found to contain 362 unique CDSs. These isolates also had a larger genome size with a higher number of functional CDSs compared to P1/7. SRD478, which was found to contain a similar number of functional CDSs compared to P1/7, was observed to contain 185 unique CDSs (Supplementary Table 1). No correlation between the number of unique genes harbored by a single isolate and ST or serotype was observed (Supplementary Table 1). The large number of unique genes and the wide variation in the number of unique genes harbored by single isolates is consistent with previous reports describing an open pan-genome characterized by a frequent gene transfer and significant genomic diversity among *S. suis* isolates (De Greeff et al., 2011; Zhang et al., 2011; Weinert et al., 2015).

Given that AMR is highly prevalent among *S. suis* isolates, the isolates in this study were screened for the phenotypic resistance and the genomes were screened for chromosomal mutations and genes conferring resistance (Supplementary Table 3). Isolates ISU2812 and ISU2912 exhibited phenotypic intermediate beta-lactam (penicillin) resistance, however no chromosomal mutations or genes conferring resistance were found (Supplementary Table 3). All isolates except P1/7 exhibited phenotypic tetracycline resistance (Supplementary Table 3). The tetracycline resistance gene *tetM* was found in SRD478, while *tetO* was found in the other tetracycline resistant isolates (Supplementary Table 3). With the exception of P1/7, ISU2660, and SRD478, all isolates exhibited phenotypic macrolide, lincosamide, and streptogramin (MLS) resistance (Supplementary Table 3). The MLS resistance gene *ermB* was found in ISU2614, ISU1606, ISU2714, ISU2514, ISU2812, and ISU2912 and both *lnuB* and *lsaE* were found in ISU2414 (Supplementary Table 3). All isolates except ISU2812 and ISU2912 exhibited phenotypic aminoglycoside resistance (Supplementary Table 3). The aminoglycoside resistance gene *ANT(9)* was identified in ISU2414. No other chromosomal mutations or genes conferring aminoglycoside resistance were found (Supplementary Table 2). ISU2414 was the only isolate to exhibit pleuromutilin resistance (tiamulin), however no genes conferring pleuromutilin resistance were found (Supplementary Table 3). With the exception of ISU2660, ISU2414, and SRD478, all isolates were phenotypically resistant to sulfonamide antibiotics (sulphadimethoxine); however, no chromosomal mutations or genes conferring resistance were found (Supplementary Table 3). No isolates were found to exhibit phenotypic resistance to phenicol, fluoroquinolone, or cephalosporin antibiotics and no chromosomal mutations or genes conferring resistance to these antibiotic classes were found (Supplementary Table 3).

Mobile genetic elements substantially contribute to evolution, adaption, and genomic diversification through horizontal gene transfer. Numerous MGEs including genomic islands and prophage insertions were observed among the genomes of the *S. suis* isolates in this study, appearing to contribute to genome plasticity and the disruption of genome synteny and plasticity (Figure 2). A complete list of all MGEs identified in the *S. suis* genomes are summarized in Supplementary Table 2. The number of prophage regions within a given genome varied from one, for isolates P1/7 and ISU2812 up to nine for ISU2912 (Table 5). Sequence analysis was used to determine if any AMR elements were located within any MGEs. Both *tetO* (DK235_04640) and *ermB* (DK235_04660) were found to be located within a genomic island for ISU1606 (Supplementary Tables 2, 3). Similarly, *tetO* (DK876_03195) and *ermB* (DK876_03150) from ISU2714 were found to be located within a genomic island (Supplementary Tables 2, 3). The *ANT(9)* (DK877_09725), *lnu(B)* (DK877_09745), and *lsaE* (DK877_09750) genes from ISU2414 are collocated within the same chromosomal region, resembling an operon structure. This region encompasses six CDSs and is not located within any of the prophage or other MGEs identified in ISU2414.

**TABLE 5:** Prophage regions identified in *S. suis* genome sequences.

**Strain**	**Region #**	**Locus^*a*^**	**Length^*b*^**	**Classification^*c*^**	**#CDSs**	**% GC content**	**Most similar phage^*d*^**	**Accession number^*e*^**
**P1/7**	1	1349343-1356912	7.5	Incomplete	9	41.3	Strept 20617 (4)	NC_023403
**ISU2614**	1	429,080-466,744	37.6	Incomplete	32	41.38	Strept SMP (7)	NC_008721
	2	829,615-844,154	14.5	Incomplete	27	38.07	Strept 20617 (4)	NC_023403
	3	1,230,839-1,271,402	40.5	Incomplete	21	35.92	Entero phiFL3A (1)	NC_013648
	4	1,441,542-1,467,871	26.3	Questionable	30	42.85	Strept 315.3 (12)	NC_004586
	5	2,191,955-2,209,769	17.8	Incomplete	22	39.34	Strept 315.2 (3)	NC_004585
**ISU1606**	1	562,081-580,311	18.2	Questionable	11	38.03	Escher RCS47/Stx2c (2)	NC_042128
	2	1,415,837-1,423,316	7.4	Incomplete	12	40.7	Strept 20617 (4)	NC_023503
**ISU2714**	1	562,204-580,434	18.2	Questionable	11	38.03	Escher RCS47/Stx2c (2)	NC_042128
	2	1,417,636-1,425,115	7.4	Incomplete	10	40.71	Strept 20617 (4)	NC_023403
**ISU2660**	1	408,070-445,755	37.6	Incomplete	32	41.39	Strept SMP (7)	NC_008721
	2	808,789-823,328	14.5	Incomplete	27	38.07	Strept 20617 (4)	NC_023403
	3	1,428,335-1,454,664	26.3	Questionable	29	42.84	Strept 315.3 (12)	NC_004586
	4	2,138,507-2,169,713	31.2	Incomplete	23	40.21	Strept 315.2 (3)	NC_004585
**ISU2514**	1	1,435,050-1,476,790	41.7	Questionable	67	41.17	Strept 315.3 (23)	NC_004586
	2	2,120,246-217,2780	52.5	Incomplete	65	40.66	Strept phiARI0462 (11)	NC_031942
	3	2,205,645-2,235,642	29.9	Incomplete	22	40.66	Lacto bIL311 (5)	NC_002670
**ISU2414**	1	429,080-466,744	37.6	Incomplete	32	41.38	Strept SMP (7)	NC_008721
	2	829,615-844,154	14.5	Incomplete	27	38.07	Strept 20617 (4)	NC_023403
	3	1,230,839-1,271,402	40.5	Incomplete	21	35.92	Entero phiFL3A (1)	NC_013648
	4	1,441,542-1,467,871	26.3	Questionable	30	42.85	Strept 315.3 (12)	NC_004586
	5	2,191,955-2,209,769	17.8	Incomplete	22	39.34	Strept 315.2 (3)	NC_004585
**ISU2812**	1	2,301,378-2,354,519	53.1	Questionable	55	40.76	Strept PH10 (27)	NC_012756
**ISU2912**	1	164,415-207,484	43	Incomplete	66	41.35	Strept phiARI0462 (12)	NC_031942
	2	316,056-347,045	30.9	Incomplete	27	40.21	Lacto bIL310 (3)	NC_002669
	3	432,867-441,801	8.9	Incomplete	9	37.76	Bacill SP 15/Stx2c 1717 (2)	NC_031245
	4	490,566-540,145	49.5	Intact	80	41.67	Strept SM1/Strept 315.3 (13)	NC_004996
	5	761,198-803,565	42.3	Questionable	69	41.23	Strept SMP (16)	NC_008721
	6	1,522,677-1,580,400	57.7	Intact	70	40.80	Lister 2389 (10)	NC_003291
	7	2,133,068-2,166,596	33.5	Incomplete	17	40.07	Strept Abc2 (2)	NC_013645
	8	2,361,356-2,393,553	32.1	Incomplete	22	40.38	Staphy SPbeta like/Salmon SJ46 (2)	NC_029119
	9	2,681,449-2,699,540	18	Incomplete	19	38.64	Strept 315.2 (4)	NC_004585
**SRD478**	1	585,583-601,969	16.3	Incomplete	9	39.42	Plankt PaV LD (1)	NC_016564
	2	2,023,417-2,038,105	14.6	Incomplete	18	40.02	Strept 315.2 (3)	NC_004585

*^*a*^Start and end basepair chromosomal location. ^*b*^Length of region depicted in kilobasepair (kb). ^*c*^Classification determined by PHASTER analysis (http://phaster.ca/). ^*d*^Most Common Phage Name (hit genes count). ^*e*^Most common phage accession number.*

Isolates ISU2614, ISU2514, and ISU2812 were observed to contain a single plasmid and SRD478 was observed to harbor four plasmids (Table 4). The plasmid harbored by ISU2614 is 4,984-bp and contains seven CDSs including *rec*, *repB*, a predicted transcriptional regulator, and four CDSs of unknown function. ISU2514 contains a 5,581-bp plasmid that encodes six CDSs including *rec*, *rep*, and four CDSs of unknown function and ISU2812 contains a similar size plasmid, 5,586-bp, which also encodes six CDSs including *rep*, and five CDSs of unknown function (Supplementary Figure 1). The first plasmid harbored by SRD478 is 15,154-bp and encodes fifteen CDSs including *parA*, *repR*, a recombinase, a kinase, a transcriptional regulator, two transporter proteins, an ATP-binding cassette-domain containing protein, a type II toxin and antitoxin (TA) system, and five additional CDSs of unknown function. A second plasmid harbored by SRD478 is 4,723-bp and contains a predicted type II toxin and antitoxin system, *rep*, and four CDSs of unknown function (Supplementary Figure 1). The third plasmid is 3,972-bp and encodes a *rep*, a bacteriocin, a bacteriocin immunity protein, and three CDSs of unknown function (Supplementary Figure 1). The fourth plasmid and smallest plasmid is 2,945-bp and contains a *rep* and two CDSs of unknown function (Supplementary Figure 1). TA systems are small genetic elements comprised of two components, a stable protein toxin and a more labile antagonistic antitoxin, and typically provide a variety of functions, such as stabilization of genomic regions, anti-addiction against similar plasmid-borne toxins, defense against phage infection, biofilm formation, control of the stress response, and bacterial persistence (Yamaguchi and Inouye, 2011; Yamaguchi et al., 2011). With the exception of bacteriocin, no other chromosomal mutations and genes conferring resistance were identified in any of the plasmids in these three strains. Bacteriocins comprise a group of structurally diverse small antimicrobial peptides, which are ribosomally synthesized by a wide range of bacteria and archaea (Dobson et al., 2012). Bacteriocin production has been considered an important and desirable trait in the selection of probiotic strains (Dobson et al., 2012; O’Connor et al., 2020). However, a bacteriocin locus was recently found encoded within an ICE of the *S. suis* prophylactic candidate strain 90-133 (Sun et al., 2019). The authors cautioned against the use of strain 90-133 due to potential transfer of bacteriocin resistance given the bacteriocin locus was found encoded within a mobile genetic element. The finding of a bacteriocin locus encoded within a relatively small mobile plasmid reported here supports the idea that bacteriocin resistance may be easily transferred and more prevalent than originally assumed.

### Capsule Loci Comparisons

Four types of capsule polysaccharide (CPS) loci were observed among the isolates in this study with the majority harboring a type 2 capsule (P1/7, ISU2614, ISU1606, ISU2714, ISU2660, ISU2514, and ISU2414) (Supplementary Figure 2). The serotype 2 strains are highly similar to each other and to reference strain P1/7, but were not identical. Sequence conservation is very high in the region spanning from the 5′ regulatory genes to the N-acetylneuraminic acid synthesis genes located near the 3′ end of the CPS locus. Within this region, identical annotated gene content compared to P1/7 was observed except for the region predicted to encode several small CDSs of unknown function between the glycosyltransferase and sialyltransferase genes (Supplementary Figure 2). While similar nucleotide sequence was observed, the annotation of P1/7 differs compared to the other serotype 2 strains. The greatest variation within the capsule region of the serotype 2 strains was seen in the region near the 3′ end containing genes predicted to encode transposases or integrases located between the N-acetylneuraminic acid synthesis and the *aroA* genes (Supplementary Figure 2). ISU2812 was identified as serotype 19 and is highly similar but not identical to the CPS locus of strain 42A, another serotype 19 strain (Supplementary Figure 2; Liu et al., 2013). As with the serotype 2 strains, gene content is conserved compared to strain 42A and the region of greatest nucleotide sequence divergence is within the mobile-element related genes near the 3′ end (Supplementary Figure 2). The CPS locus of ISU2912 was identified as a NCL serotype 1-3, originally described by Zheng et al. (2015) (Supplementary Figure 2). Sequence comparisons with other NCL1-3 strains showed similarity in gene content, with the exception that ISU2912 contains transposases not present in the NCL1-3 strains from Zheng et al. (2015) (Supplementary Figure 2). These are the transposases located between the sugar epimerase gene and the sugar dehydrogenase gene, and the 4 genes immediately downstream of the nucleotidyl transferase gene (1 unknown function, 3 transposase/integrase genes) (Supplementary Figure 2; Zheng et al., 2015). The CPS locus of SRD478 does not match any published serotypes; however, a BLASTn search revealed a high similarity to CPS loci from four non-typeable *S. suis* isolates (accession numbers KX870048, KX870054, KX870055, and KX870059) (Supplementary Figure 2).

**FIGURE 4:**
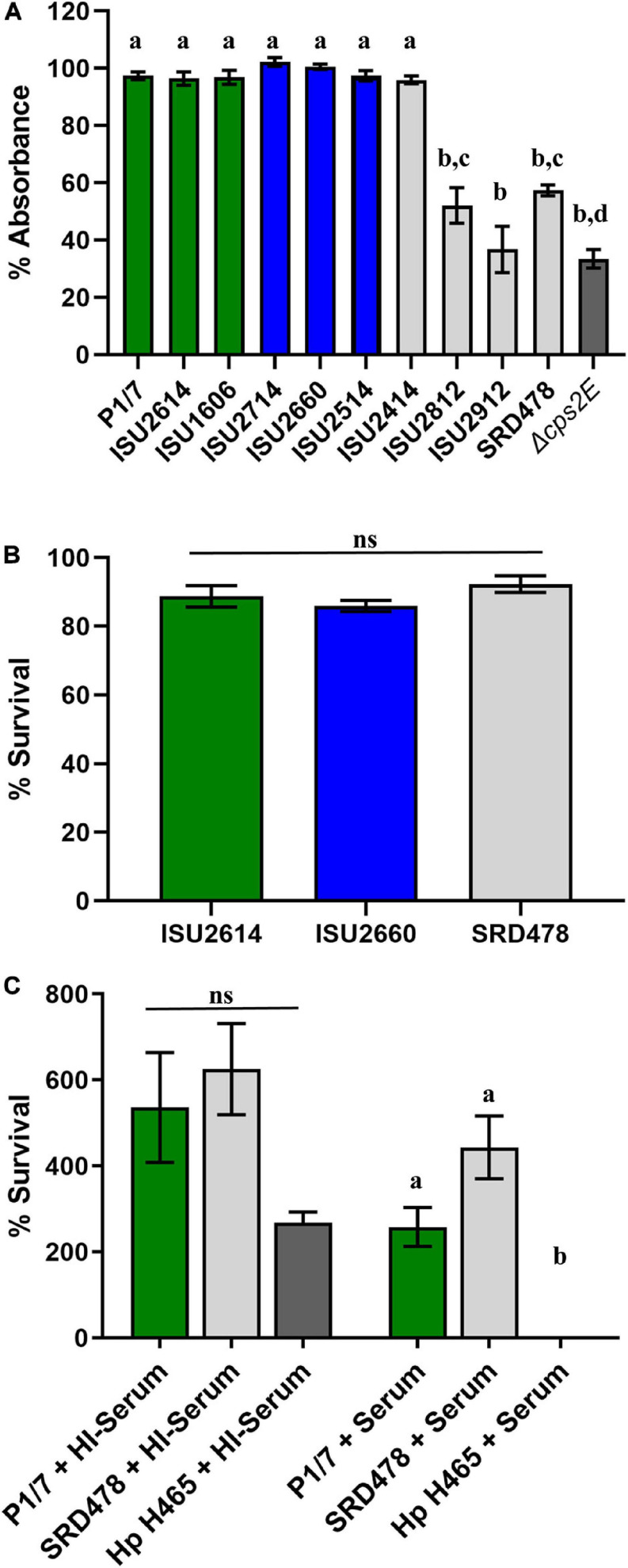
Comparison of phenotypes related to capsule production. **(A)** Bacterial hydrophobicity assay. Relative hydrophobicity of *S. suis* isolates were determined by measuring their absorption (*y*-axis) to n-hexadecane. *S. suis* P1/7 Δ*cps2E*, a capsule deletion mutant, was used as a control. **(B)** Whole blood sensitivity assay. *S. suis* isolates were incubated with swine whole blood for 1 h and enumerated to determine the percent viable bacteria (*y*-axis) expressed. **(C)** Serum sensitivity assay. Bacteria were incubated with guinea pig serum (+Serum) or heat-inactivated guinea pig serum (HI + Serum) and enumerated to determine the percent survival (*y*-axis) calculated as the proportion of treated samples to untreated samples. Serum-sensitive *Glasserella parasuis* H465 (Hp H465) was used as a control. Bars represent means ± SEM from three independent experiments. Bar color reflective of virulence categorization (green: highly virulent; blue: moderately virulent; light gray: non-virulent) or control (dark gray: *S. suis* P1/7 Δ*cps2E*). Data was analyzed using a one-way ANOVA with a Tukey’s post-test (GraphPad Prism 8.3.0). Groups with different letter designations were significantly different (*p* < 0.05); ns, not statistically significantly different.

While a variety of virulence factors have been described for *S. suis*, CPS is perhaps considered the most important (Charland et al., 1998; Fittipaldi et al., 2012; Roy et al., 2015; Zhao et al., 2015; Segura et al., 2017). Therefore, the capsule production ability among the *S. suis* isolates was evaluated using *in vitro* assays, which have been previously used to demonstrate CPS production (Bonifait et al., 2010; Roy et al., 2015; Auger et al., 2018). All of the isolates harboring a type 2 capsule (P1/7, ISU2614, ISU1606, ISU2714, ISU2660, ISU2514, and ISU2414) exhibited a low surface hydrophobicity (higher percent retained in the aqueous phase), reflective of high capsule production, and significantly different compared to the P1/7 capsule mutant Δ*cps2E* (Figure 4A). In contrast, ISU2812 (serotype 19), ISU2912 (serotype 1-3), and SRD478 (undefined/non-typeable) exhibited a high surface hydrophobicity, indicating reduced CPS production, compared to the type 2 capsule isolates (Figure 4A). ISU2912 was the only isolate that exhibited no significant difference in surface hydrophobicity compared to the P1/7 capsule mutant Δ*cps2E* (Figure 4A). The ability to survive incubation in whole blood was tested for one isolate from each swine virulence category. Out of the isolates that exhibited high surface hydrophobicity, ISU2912 would have been the best choice to further test for the ability to survive in both whole blood and serum. However, ISU2912 was observed to have a variety of traits, including genomic size, sequence diversity, and growth medium conditions, which differed from the other isolates and was therefore excluded from consideration in an effort to minimize confounders. Out of the isolates that exhibited high surface hydrophobicity, SRD478 was chosen to test for the ability to survive in both whole blood and serum. No significant difference in the ability to survive incubation in whole blood was observed for ISU2614 (highly virulent), ISU2660 (moderately virulent), and SRD478 (non-virulent) (Figure 4B). While a reduction in the percent survival was observed after incubation in serum compared to heat-inactivated serum, no significant difference in the percent survival between P1/7 (highly virulent) and SRD478 (non-virulent) was observed (Figure 4C).

It has been previously demonstrated that CPS production results in low surface hydrophobicity (Bonifait et al., 2010; Roy et al., 2015; Auger et al., 2018). While the high surface hydrophobicity exhibited by ISU2812, ISU2912, and SRD478 could suggest a decreased ability to produce a capsule, the ability of SRD478 to survive in both whole blood and serum suggest that these isolates may have the ability to produce a capsule, albeit with different functional characteristics. The lack of significant difference between P1/7 (highly virulent) and SRD478 (non-virulent) in the ability to survive in both whole blood and serum suggest that any differences or functional characteristics between their capsules does not correlate with their respective capacity to cause disease in swine.

### Comparison of *in vitro* Growth and Virulence-Associated Phenotypes

**FIGURE 5:**
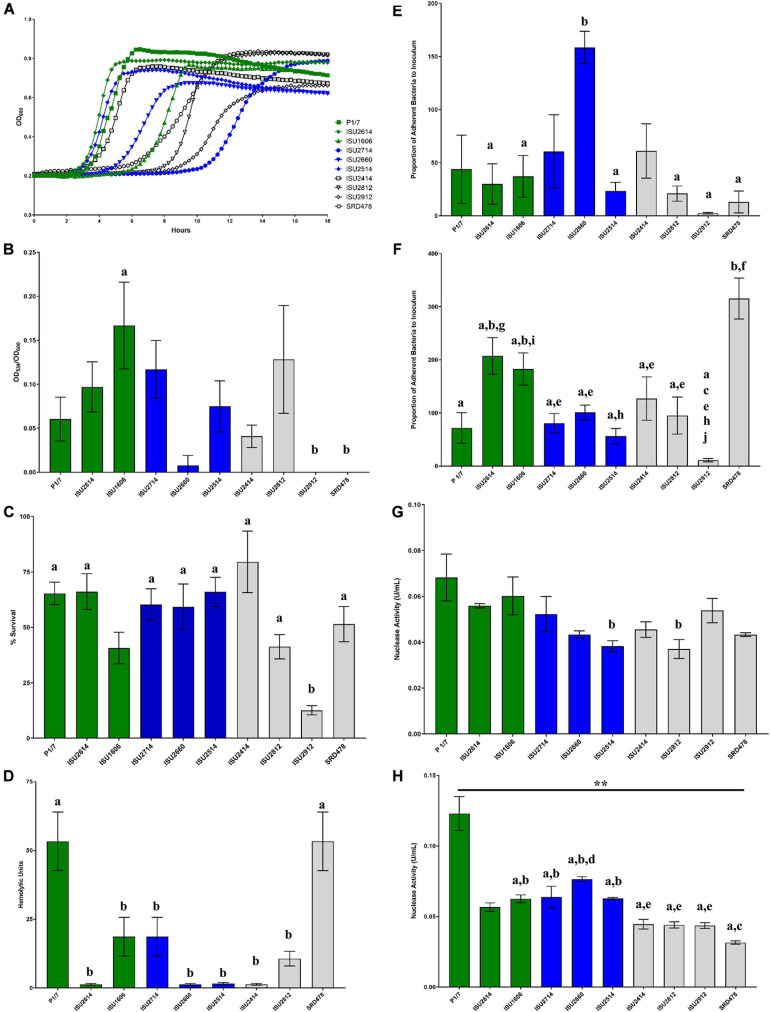
Comparison of *in vitro* growth and virulence-associated phenotypes. **(A)** Growth dynamics of *S. suis* isolates. Isolates were cultivated at 37°C for 24 h. Growth was measured by OD_600_ every 15 min. All data points represent averages obtained from three independent experiments. **(B)** Biofilm formation by *S. suis* isolates. *S. suis* isolates were cultivated statically for 24 h in microtiter plates. Growth was then measured by OD_600_ followed by quantification crystal violet staining by OD_538_. Biofilm mass (*y*-axis) is expressed as the OD_538_ normalized to OD_600_. **(C)** Oxidative stress assay. *S. suis* isolates were cultivated until exponential phase and divided into treated (10 mM H_2_O_2_) and untreated cultures (H_2_0), incubated at 37°C for 15 min followed by addition of 10 μg/mL catalase. Bacteria were enumerated to determine the percent survival (*y*-axis) calculated as the proportion of treated samples to untreated samples. **(D)** Hemolysis assay. Hemolytic activity of supernatants collected from *S. suis* isolates. **(E)** Adherence of *S. suis* isolates to BEAS-2B cells. Adherence (*y*-axis) to BEAS-2B cells (human lung/bronchus epithelial cell line) is expressed as the proportion of bacteria in the original inoculum found to be adherent after a 2-h incubation period. **(F)** Adherence of *S. suis* isolates to macrophages. Adherence (*y*-axis) to J774A.1 cells (murine macrophage-like cell line cell line) is expressed as the proportion of bacteria in the original inoculum found to be adherent after a 2-h incubation period. **(G)** Cell-Associated Nuclease Activity. Cell-associated nuclease activity for *S. suis* isolates was measured using whole-cell FRET assay. **(H)** Secreted Nuclease Activity. Secreted nuclease activity for *S. suis* isolates was measured using extracellular FRET assay. Bars **(B–H)** represent means ± SEM from three independent experiments. Bar color reflective of virulence categorization (green: highly virulent; blue: moderately virulent; light gray: non-virulent). Data **(B–H)** was analyzed using a one-way ANOVA with a Tukey’s post-test (GraphPad Prism 8.3.0). Groups with connecting lines were significantly different (*p* < 0.0003). Groups with different letter designations were significantly different (*p* < 0.05).

Given the lack of correlation between capsule type and the capacity to cause disease in swine, we further tested the *S. suis* isolates in a variety of *in vitro* assays routinely used to measure virulence capacity. Differences in growth rate dynamics of *S. suis* isolates were measured and revealed a wide range of growth rates among the isolates (Figure 5A). Overall, no correlation between the growth rate dynamics of the *S. suis* isolates and their capacity to cause disease in swine was observed (Figure 5A). It has been reported that biofilm formation in *S. suis* causes a reduction in virulence due to the downregulation of virulence factors, such as CPS (Wang et al., 2011). Biofilm formation among the *S. suis* isolates was quantified by standard microtiter crystal violet assays (Figure 5B). While significant differences in biofilm formation were observed among some of the isolates, no correlation between biofilm formation by the *S. suis* isolates and their capacity to cause disease in swine was observed (Figure 5B). Moreover, no correlation between biofilm formation by the *S. suis* isolates and their surface hydrophobicity was observed (Figures 4A, 5B). Next, the ability to survive oxidative stress, to produce hemolytic activity, to adhere to either BEAS-2B or J774A.1 cells, as well as the ability to produce cell-associated or secreted nuclease activity among the *S. suis* isolates was measured (Figures 5C–H). Similar to the growth rate dynamics and biofilm formation capacity among the *S. suis* isolates, significant differences in each of these virulence-associated phenotypes were observed among some of the isolates; however, there was no correlation between *in vitro* virulence-associated phenotypes by the *S. suis* isolates and their capacity to cause disease in swine (Figures 5C–H).

### Virulence-Associated Gene Comparisons

**FIGURE 6:**
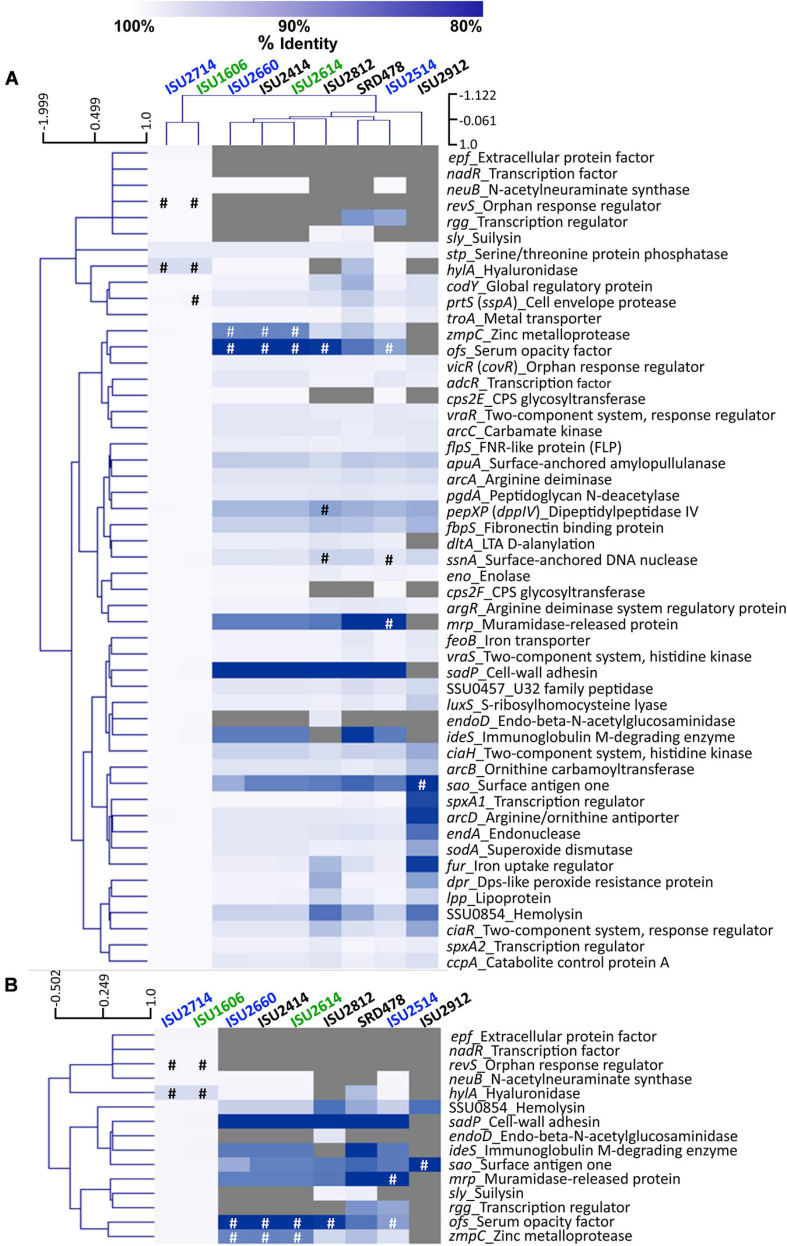
Hierarchical cluster heatmap displaying the relatedness of *S. suis* isolates based on the nucleotide percent identity of analyzed virulence genes. **(A)** Heatmap based on nucleotide percentage identity for all analyzed virulence genes. Heatmap generated from nucleotide percentage identity was converted into a distance matrix and clustered by means of complete hierarchical clustering based on Pearson correlation distance for both genes and isolates. **(B)** Heatmap based on nucleotide percentage identity for selected genes with high sequence divergence. Heatmap generated from nucleotide percentage identity was converted into a distance matrix and clustered by means of complete hierarchical clustering based on Pearson correlation distance for genes only. Isolates names are provided at the top of the heat map and gene names are provided at the right side of heat map. Font color used for isolate names color reflective of virulence categorization (green: highly virulent; blue: moderately virulent; black: non-virulent). The percent identity of analyzed genes (rows) from each isolate (columns) is represented using the color scale at top, while genes not present within a strain are indicated by gray. Annotated pseudogenes are indicated by # (white or black). Dendrograms are on the left side and on top of the heat map.

To further examine genomic differences that could have influenced the swine virulence capacity of the *S. suis* isolates, we compared the nucleotide sequences of fifty-one virulence-associated genes, which were included in a comprehensive review (Fittipaldi et al., 2012). The percent identity for each gene was determined for each isolate relative to the P1/7 ortholog with the following exceptions: *hylA*, *revS*, *stp*, *vraR*, and *vraS*. These exceptions were based on choosing a reference gene sequence in which functional characterization had previously been reported or due to annotation of the P1/7 gene as a pseudogene. Hierarchical clustering analysis of the nucleotide percent identity of analyzed virulence genes revealed two general observations. First, a correlation between ST and the presence of genes encoding known virulence factors, and the nucleotide identity among those genes, was observed (Figure 6A). The highest sequence identity among the analyzed genes was observed in the genes from isolates ISU1606 and ISU2714. Similar to P1/7, both of these isolates are ST-1 (Figure 6A). Second, no clear correlation between the capacity to cause disease in swine and the presence of genes encoding known virulence factors, and the nucleotide identity among those genes, was found (Figure 6A). For example, while the highest sequence divergence in the genes analyzed was observed from the non-virulent isolate ISU2912, similarly high sequence divergence was not noted for the other non-virulent isolates (ISU2414, ISU2812, and SRD478) (Figure 6A). While a high sequence identity among the analyzed genes was observed for the highly virulent ISU1606, high sequence identity among the analyzed genes was not observed for ISU2614, also categorized as highly virulent. Some of the analyzed ISU2614 genes with the lowest sequence identity include *sadP* with 77% identity, *ideS* with 87.5% identity, and *mrp* with 87.8% identity compared to the corresponding P1/7 orthologs (Figures 6A,B). ISU2614 genes *ofs*, with 75.9% identity, and *zmpC*, with 75.9% identity, are both predicted pseudogenes Figures 6A,B. Suilysin gene *sly* from ISU2714 and ISU1606 was 100% identical to *sly* from P1/7 and *sly* from the non-virulent isolates ISU2812 and SRD478 were 99.6% and 99.2% identical to *sly* from P1/7. However, *sly* was absent from all other isolates (ISU2660, ISU2414 ISU2614, ISU2514, and ISU2912) (Figure 6A).

The closest correlation between the capacity to cause disease in swine and the presence of genes encoding known virulence factors, or their nucleotide identity, was observed for the genes *neuB*, *ciaR*, *codY*, and SSU0854 encoding a hemolysin. Specifically, *neuB* was present in ISU2414, but absent from the other non-virulent isolates ISU2812, ISU2912, and SRD478 (Figure 6A). A similar sequence identity was observed between P1/7 and ISU2414 for *ciaR*, *codY*, and SSU0854 encoding a hemolysin, while a low sequence identity was observed between P1/7 and the other non-virulent isolates ISU2812, ISU2912, and SRD478 (Figure 6A). Therefore, no clear correlation between the presence of genes encoding known virulence factors, or their nucleotide identity, and the capacity to cause disease in swine was identified.

Overall the highest sequence identity among the analyzed genes was observed in the genes from isolates ISU1606, categorized as highly virulent, and ISU2714, categorized as moderately virulent (Figure 6A). As mentioned, ISU1606 and ISU2714 harbor the same ST as P1/7 (ST-1). The *hylA* gene encoding hyaluronidase from both ISU1606 and ISU2714 was found to have the most sequence divergence with 97.3% identity and is a predicted pseudogene in ISU1606 and ISU2714 (Figure 6A). The *prtS* (*sspA*) gene encoding a cell envelope protease is also a predicted pseudogene in ISU1606 (Figure 6A). Another predicted pseudogene in ISU1606 and ISU2714 is *revS*, which is also annotated as a pseudogene in P1/7 (Figures 6A,B). Since *revS* is annotated as a pseudogene in P1/7, the *revS* gene from *S. suis* 05ZHY33 (Wu et al., 2009) was used as the comparison reference. Previous studies reported *revS* to be a 519-bp gene encoding a 173-amino acid protein (De Greeff et al., 2002; Wu et al., 2009). The *revS* gene for ISU1606 and ISU2714 is predicted to be 821-bp and 815-bp for P1/7. The 5′ end of *revS* for all three isolates is 100% identical to the 519-bp *revS* gene from *S. suis* 05ZHY33, which was used as reference. Therefore, it is possible that the first 519-bp of the *revS* gene from ISU1606, ISU2714, and P1/7, could encode a functional RevS protein. *revS* orthologs were not identified in any of the other isolates analyzed (Figures 6A,B). Similarly, *nadR* and *epf* from ISU1606 and ISU2714 were 99.9% and 100% identical to their respective P1/7 orthologs and were not identified in any of the other isolates (Figures 6A,B).

**FIGURE 7:**
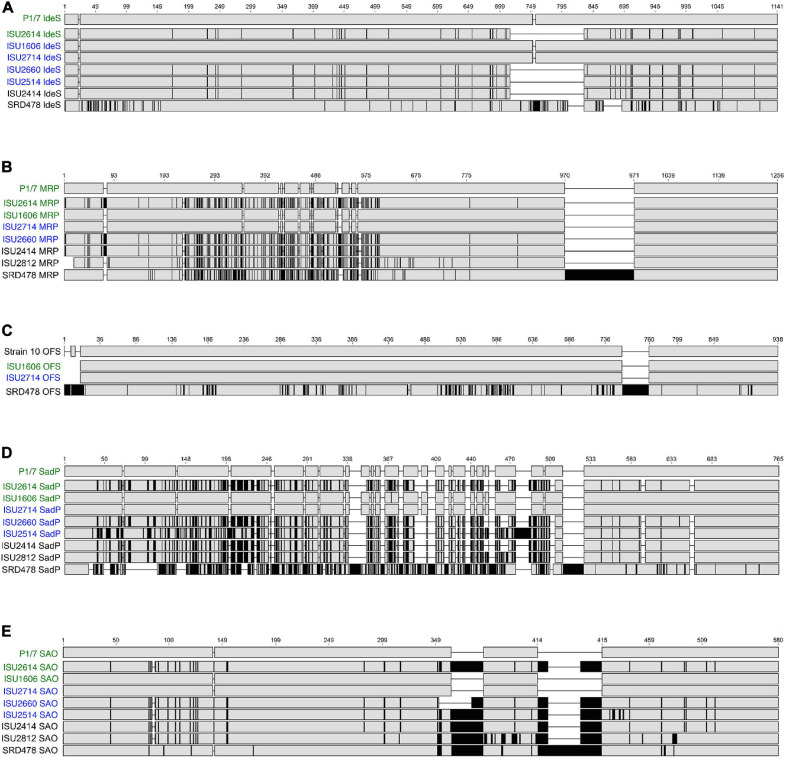
Protein sequence alignments. Gene sequences were identified by BLASTn, translated, and multiple alignments of the protein sequences were created using MAFFT. Gray regions indicate identical amino acids, black regions indicate amino acid sequence differences compared to the reference (top) sequence. **(A)** Multiple alignment of IdeS (immunoglobulin M-degrading enzyme); P1/7 IdeS used as reference sequence. **(B)** Multiple alignment of MRP (muramidase-released protein); P1/7 MRP used as reference sequence. **(C)** Multiple alignment of OFS (serum opacity factor); Strain 10 OFS used as reference sequence. **(D)** Multiple alignment of SadP (cell wall adhesin); P1/7 SadP used as reference sequence. **(E)** Multiple alignment of SAO (surface antigen one); P1/7 SAO used as reference sequence.

Due to the high degree of nucleotide divergence observed for some of the genes listed in Figure 6B, protein sequence alignments were generated to explore the amino acid variation. IdeS is a *S. suis* immunoglobulin M-degrading enzyme, first described by Seele et al. (2013). The predicted amino acid sequence contains a Mac-1 domain and is similar to the IdeS, also known as Mac-1, a *S. pyogenes* endopeptidase with specificity for IgG (Agniswamy et al., 2004, 2006; Wenig et al., 2004). IdeS from P1/7, ISU1606, and ISU2714 is identical and is 1,141 amino acids (AA) in length (Figure 7A). IdeS from ISU2614, ISU2660, and ISU2514 and ISU2414 is shorter with a predicted 1,027 AA length, mainly due to a 114 AA deletion corresponding to amino acid residues 717-830 of P1/7 IdeS (Figure 7A). There are 151 AA differences between IdeS from ISU2614, ISU2660, and ISU2414 compared to P1/7 IdeS and 150 AA differences between IdeS from ISU2514 and P1/7 (Figure 7A). These changes are observed throughout the protein sequences (Figure 7A). The greatest amino acid divergence was observed in IdeS from SRD478, predicted to be 1092 AA in length and contain 170 differences compared to P1/7 IdeS (Figure 7A). Many of these differences are located with the N-terminus region as well two small deletion regions of 25 AA and 29 AA that correspond to 806-830 AA and 86-891 AA in P1/7 IdeS (Figure 7A).

While a search failed to identify a corresponding *ideS* ortholog in ISU2812 and ISU2912, further inspection of the genomic region within these isolates corresponding to the *ideS* location in the P1/7 genome revealed a CDS annotated as a hypothetical protein containing a Mac-1 domain in both ISU2812 (A7J09_05595) and ISU2912 (A7J10_03470). Both CDSs are 2,727-bp and are 99.4% identical to each other. The predicted amino acid sequence for both is 908 AA and has 99.1% similarity to each other. The predicted amino acid sequence for the CDS from ISU2812 (A7J09_05595) only has 28.7% similarity to P1/7 IdeS, with the highest level of similarity located within the cysteine proteinase/Mac-1 domain region.

Muramidase-released protein is a muraminidase-released protein that has been characterized as associated with the cell wall of *S. suis* via an LPxTG motif as well as released into culture supernatants (Vecht et al., 1991). MRP is often reported as an epidemic infection marker for highly virulent strains in Europe and Asia (Vecht et al., 1996; Silva et al., 2006; Rehm et al., 2007; Wei et al., 2009; Onishi et al., 2012; Maneerat et al., 2013; Li et al., 2017). The amino acid sequence of MRP from P1/7, ISU1606, and ISU2714 were identical, 1,256 AA in length, and contained a signal peptide domain followed by several repeat MucBP (Mucin-Binding Protein) domains, followed by a LPXTG cell wall anchor domain (Figure 7B). MRP from ISU2614 and ISU2660, and ISU2414 was predicted to be 1,268 AA in length and the protein sequence from these isolates differed from P1/7 MRP by 177 AA. MRP from ISU2812 was predicted to be 1,242 AA in length and contained 183 differences compared to P1/7 MRP (Figure 7B). The predicted AA sequence for MRP from SRD478 was 1,391 AA and differed from P1/7 MRP by 338 AA. MRP from SRD478 contained a large 136 AA insert within a MucBP domain corresponding to 970-971 AA in P1/7 MRP (Figure 7B). Many of the AA differences in the MRP from isolates ISU2614, ISU2660, ISU2414, ISU2812, as well as in SRD478 compared to P1/7 MRP were located upstream of the first MucBP domain (Figure 7B). The observed variation in size of MRP from the isolates analyzed was not surprising given that MRP size variants have been previously described (Silva et al., 2006). MRP from ISU2514 contained a frameshift mutation and was annotated as a pseudogene.

The *ofs* gene encodes for the *S. suis* serum opacity factor (OFS) protein. The predicted OFS protein structure has been described to contain several structural features of a MSCRAMMs (microbial surface components recognizing adhesive matrix molecules), such as a putative N-terminal signal sequence, a large N-terminal domain including a proline-rich region, repetitive sequence elements, and a C-terminal LPXTG anchor motif (Baums et al., 2006). Several allelic variants of OFS have been previously reported (Takamatsu et al., 2008). Due to frameshift mutations in P1/7, ISU2414, ISU2614, ISU2812, and ISU2660, and a truncation in ISU2514, the *ofs* gene was annotated as a pseudogene.

Protein alignments were generated using OFS from *S. suis* Strain 10 as the reference since it has been functionally characterized and demonstrated to serve a role in virulence (Baums et al., 2006). OFS from Strain 10 is predicted to be 938 AA in length, while OFS from ISU1606 and ISU2714 is predicted to be 930 AA (Figure 7C). OFS from ISU1606 and ISU2714 differs from Strain 10 OFS by 1 AA, which happens to be the start codon for ISU1606 and ISU2714. The difference in predicted length is due to annotation differences in which the annotation for Strain 10 uses an upstream start codon compared to the start codon used in the annotation of ISU1606 and ISU2714. The nucleotide sequence upstream of the annotated *ofs* CDS for ISU1606 and ISU2714 is identical to the corresponding region in Strain 10 and the corresponding start codon used in the annotation of Strain 10 is inframe with the annotated *ofs* CDS for ISU1606 and ISU2714. Therefore, despite the predicted length difference, OFS from Strain 10, ISU1606, and ISU2714 is likely to be highly similar. OFS from SRD478 is predicted to be 989 AA in length and was observed to contain the greatest amino acid divergence with 183 differences compared to Strain 10 OFS (Figure 7C). Many of these differences are located in two regions. First, several small insertions are located within the first 22 AA of the N-terminal signal peptide and the second location is a 37 AA insertion corresponding to 759 and 760 of Strain 10 OFS (Figure 7C).

SadP (streptococcal adhesin P) has been demonstrated to mediate the binding of *S. suis* to galactosyl-α1–4-galactose (Galα1–4Gal)-containing host receptors (Kouki et al., 2011). Substantial nucleotide sequence variation in the *sadP* gene and structural variants for SadP have been described (Ferrando et al., 2017). SadP from P1/7, ISU1606, and ISU2714 is 765 amino acids (AA) in length and SadP from P1/7 and ISU2714 is identical, while SadP from ISU1606 differs from P1/7 SadP by 1 AA (Figure 7D). A high degree of amino acid divergence was observed for SadP from the other isolates (Figure 7D). SadP from ISU2614, ISU2660, and ISU2414 is shorter with a predicted 714 AA in length (Figure 7D). There are 241 AA differences between SadP from ISU2614 and ISU2414 compared to P1/7 SadP and 242 AA differences between SadP from ISU2660 and P1/7 (Figure 7D). SadP from ISU2514 is predicted to be 747 AA in length and contain 254 differences compared to P1/7 SadP (Figure 7D). The predicted amino acid sequence for SadP from ISU2812 is 728 AA and differs from P1/7 SadP by 235 AA. SRD478, predicted to be 769 AA in length and differs from P1/7 SadP by 422 AA, which includes a 26 AA insert corresponding to 524-525 of P1/7 SadP (Figure 7D). Kouki et al. (2011) reported that the central portion of the protein, particularly amino acids 31-328, are critical for ligand binding to Galα1–4Gal-containing host receptors. Given that the majority of the numerous AA changes observed in SadP from ISU2614, ISU2660, ISU2514, ISU2414, ISU2812, and SRD478 are located within this region, it is possible that the variation could impact binding affinity, ligand septicity, or both.

Surface antigen one (SAO) was originally identified by a phage display library screen utilizing convalescent swine sera and has been reported to contain a LPXTG anchor motif and is consequently anchored to the cell-wall peptidoglycan by sortase A (Li et al., 2007; Zhang et al., 2008). While it has not previously been associated with virulence, it has been evaluated as a protective immunogen (Li et al., 2007; Zhang et al., 2008; Hsueh et al., 2014, 2017; Roy et al., 2014). To date, three allelic variants have been reported with *sao*-M, encoding an amino acid sequence 580 AA in length, as the most prevalent (Feng et al., 2007). SAO from P1/7, ISU1606, and ISU2714 are identical and are 580 AA in length (Figure 7D). The amino acid sequence contains an FctA domain, found in the major pilin of from *Streptococcus pyogenes* and other fibronectin- and collagen-binding proteins, located between AA524-525 of P1/7 SAO. SAO from ISU2614, ISU2514, ISU2414, and ISU2812 is longer with a predicted 639 AA length (Figure 7E). There are 92 AA differences between SAO from ISU2614 compared to P1/7 SAO, 99 AA differences between SAO from ISU2514 and P1/7 SAO, and 91 AA differences between SAO from ISU2414 and P1/7 SAO (Figure 7E). The greatest amino acid divergence was observed in SAO from ISU2812, which differs from P1/7 SAO by110 AA (Figure 7E). SAO from SRD478 is predicted to be 670 AA in length and contain 105 differences compared to P1/7 SAO (Figure 7E). The *sao* gene in ISU2912 (A7J10_07360, A7J10_07370) is interrupted by a transposase (A7J10_07365). The alignment revealed two areas of insertions downstream of the FctA domain that are different lengths for different isolates (Figure 7E). The first insertion location corresponds to AA363-364 in P1/7 SAO (Figure 7E). ISU2614, ISU2514, ISU2414, ISU2812, and SRD478 contain a 30 AA insertion and ISU2660 contains an 11 AA insertion at this location (Figure 7E). The second insertion location corresponds to positions 414-415 in P1/7 SAO (Figure 7E). ISU2614, ISU2660, ISU2514, ISU2414, and ISU2812 contain a 30 AA insertion and SRD478 contains a 60 AA insertion at this location (Figure 7E).

Due to the high degree of nucleotide divergence observed for the fifty-one previously reported virulence-associated genes (Figure 6A), we performed an additional search for orthologs that were found in any one of the three isolates categorized as highly virulent (P1/7, ISU2614, or ISU1606) and not found in any of the isolates categorized as non-virulent (ISU2414, ISU2812, ISU2912, and SRD478). This analysis identified 42 CDSs including one predicted DNA binding protein and three predicted transcriptional regulators (Supplementary Table 1). Unfortunately, the majority of the other CDSs were predicted to be uncharacterized hypothetical proteins (Supplementary Table 1). A reciprocal search for orthologs was then performed in which a search for orthologs found in any one of the isolates categorized as non-virulent (ISU2414, ISU2812, ISU2912, or SRD478) and not found in any of the isolates categorized as highly virulent (P1/7, ISU2614, and ISU1606). This analysis identified 1,425 CDSs including 13 predicted DNA binding proteins and 63 predicted transcriptional regulators (Supplementary Table 1). The much larger number of CDSs identified from this search is likely reflective of the larger genome size of isolates ISU2812 and ISU2912. While this analysis failed to pinpoint any specific CDSs that likely function as potential virulence factors, it does provide a reliable one-to-one assignment of specific genes of interest that could prove useful in future allelic replacement and/or functional genomic studies.

## Conclusion

In summary, a spectrum of virulence phenotypes was observed among the nine United States *S. suis* isolates following intranasal challenge in pigs. Comparative genomic analysis of the genomes revealed a high degree of similarity among isolates P1/7, ISU1606, and ISU2714 from a variety of different assessments, while extensive genetic variation was observed among the other isolates. Genomic analysis revealed a bacteriocin locus encoded within a relatively small mobile plasmid, as well as numerous other chromosomal MGEs within these *S. suis* genomes, some of which contained AMR genes. While virulence mechanisms utilized by *S. suis* are not fully understood, a variety of different functional characteristics of *S. suis* isolates have been hypothesized to serve a role in virulence. In this study, no correlation between the characteristics evaluated here, such as genome size, serotype, ST, *in vitro* virulence-associated phenotypes and the capacity to cause disease in swine was observed. Focusing in on the *in vitro* virulence-associated phenotypes, results from cell-surface hydrophobicity, biofilm capacity, and the ability to survive in both whole-blood and serum, suggest that any differences or functional characteristics between *S. suis* capsules does not correlate with their respective capacity to cause disease in swine. It is important to note that while no correlation between the capacity to cause disease in swine and the presence of genes encoding known virulence factors was observed, the differential expression of any one or a combination of the genes encoding known virulence factors could strongly influence the capacity of an isolate to cause disease in swine. Despite not observing a correlation between the capacity to cause disease in swine and the presence of genes encoding known virulence factors, numerous CDSs were identified from the search for orthologs present in any one of the three isolates categorized as highly virulent and not found in any of the isolates categorized as non-virulent as well as the reciprocal ortholog search. Our hope is that the assembly and annotation of these genomes, coupled with the comparative genomic analyses reported in this study provides a framework for future allelic replacement and/or functional genomic studies investigating genetic characteristics that underlie and influence the phenotypic differences among these isolates.

## Data Availability Statement

The datasets presented in this study can be found in online repositories. The names of the repository/repositories and accession number(s) can be found in the article/Supplementary Material.

## Ethics Statement

The animal studies were conducted in accordance with the recommendations in the Guide for the Care and Use of Laboratory Animals of the National Institutes of Health. The animal experiments were approved by the USDA-National Animal Disease Center’s Institutional Animal Care and Use Committee (protocol #2724).

## Author Contributions

TN conceived and designed the experiment. TN, UW, SH, SB, and SS performed the experiments. UW, TA, SZ, IG, ME, SH, SB, DB, and SS analyzed the data. TN, UW, TA, ME, SH, SB, DB, and SS contributed reagents, materials, and analysis tools and wrote the manuscript. All authors gave approval of the final version to be published and agreed to be accountable for all aspects of the work.

## Conflict of Interest

The authors declare that the research was conducted in the absence of any commercial or financial relationships that could be construed as a potential conflict of interest.

## Footnotes

1 https://www.pacb.com/products-and-services/analytical-software/smrt-analysis/

2 https://github.com/JCVenterInstitute/PanACEA

3 http://www.genomicepidemiology.org/

4 https://card.mcmaster.ca/home

## Supplementary Material

Supplementary Figure 1 Map of plasmids detected in *S. suis* isolates. Plasmid name, accession number, and size (bp) is indicated for each plasmid (center). Arrows indicate orientation of annotated CDSs; blue arrows represent CDSs with predicted functions based on sequence homology along with indicated names. Gray arrows represent predicted CDSs of unknown function. **(A)** pISU2614. **(B)** pISU2514. **(C)** pISU2812. **(D)** pSRD487_1. **(E)** pSRD487_2. **(F)** pSRD487_3. **(G)** pSRD487_4.

Supplementary Figure 2 Comparison of capsule loci. Colored arrows represent the genes present within the CPS locus of the representative serotypes and *S. suis* isolates (left). Predicted function is indicated by the arrow color, as shown at the bottom. ISU2614 was chosen as a representative of the serotype 2 strains, ISU2812 is serotype 19, ISU2912 is serotype NCL1-3 and the serotype of SRD478 is undefined. Specific regions of greatest nucleotide sequence divergence observed within a specific serotype is indicated by brackets and letters.
