# Supplementary figures and images for: Comparative Virulence and Genomic Analysis of Streptococcus suis Isolates
Source: Front Microbiol. 2021 Jan 26;11:620843. doi: 10.3389/fmicb.2020.620843 (PMC7870872; doi:10.3389/fmicb.2020.620843)

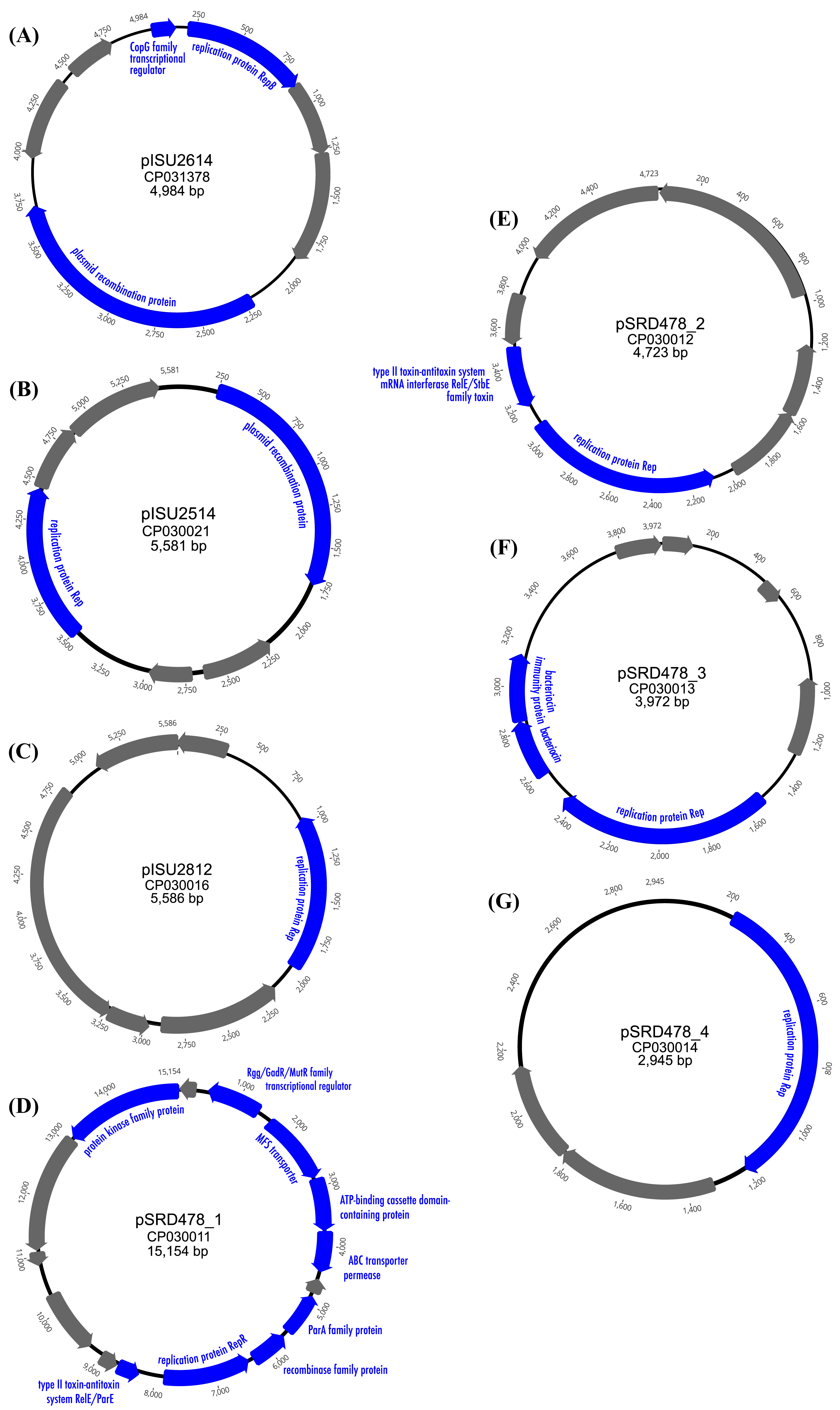

Supplement: Supplementary Figure 1 — Map of plasmids detected in S. suis isolates. Plasmid name, accession number, and size (bp) is indicated for each plasmid (center). Arrows indicate orientation of annotated CDSs; blue arrows represent CDSs with predicted functions based on sequence homology along with indicated names. Gray arrows represent predicted CDSs of unknown function. (A) pISU2614. (B) pISU2514. (C) pISU2812. (D) pSRD487_1. (E) pSRD487_2. (F) pSRD487_3. (G) pSRD487_4. [file Image_1.TIF]

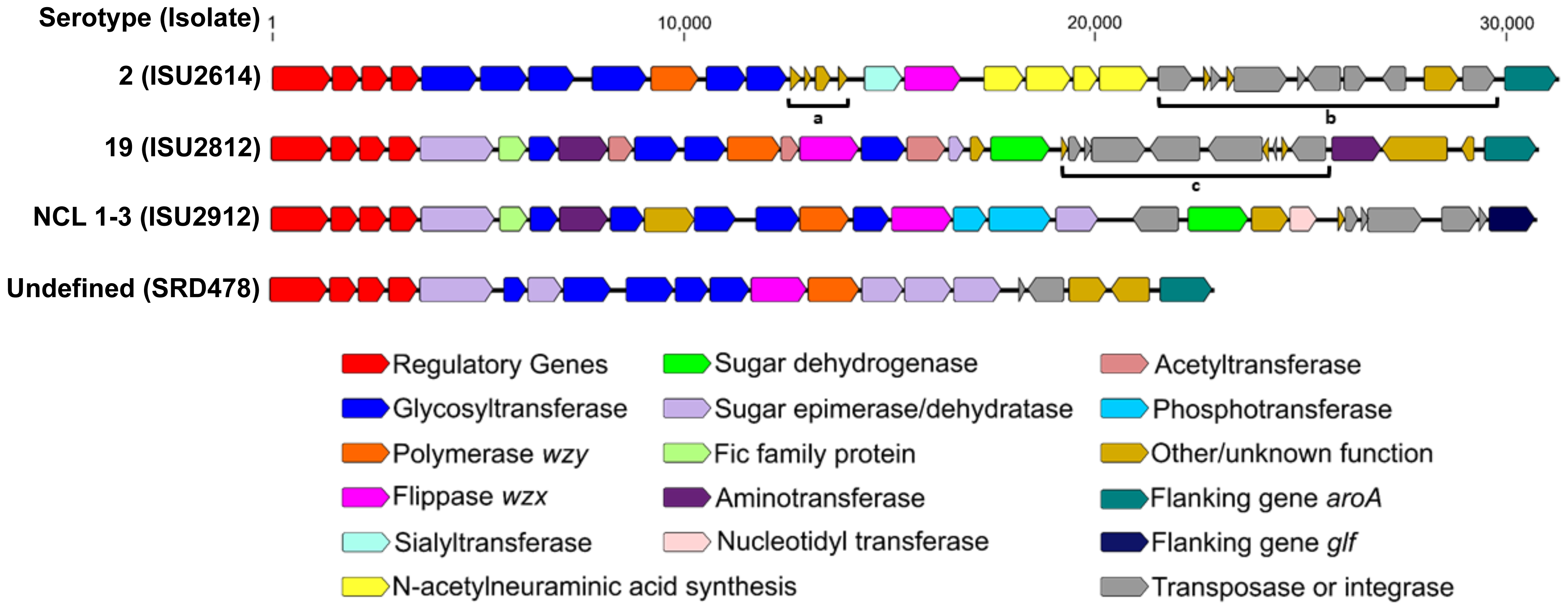

Supplement: Supplementary Figure 2 — Comparison of capsule loci. Colored arrows represent the genes present within the CPS locus of the representative serotypes and S. suis isolates (left). Predicted function is indicated by the arrow color, as shown at the bottom. ISU2614 was chosen as a representative of the serotype 2 strains, ISU2812 is serotype 19, ISU2912 is serotype NCL1-3 and the serotype of SRD478 is undefined. Specific regions of greatest nucleotide sequence divergence observed within a specific serotype is indicated by brackets and letters. [file Image_2.TIF]
